# Neofunctionalization of Chromoplast Specific Lycopene Beta Cyclase Gene (*CYC-B*) in Tomato Clade

**DOI:** 10.1371/journal.pone.0153333

**Published:** 2016-04-12

**Authors:** Vijee Mohan, Arun Pandey, Yellamaraju Sreelakshmi, Rameshwar Sharma

**Affiliations:** Repository of Tomato Genomics Resources, Department of Plant Sciences, University of Hyderabad, Hyderabad, India; University of Tsukuba, JAPAN

## Abstract

The ancestor of tomato underwent whole genome triplication ca. 71 Myr ago followed by widespread gene loss. However, few of the triplicated genes are retained in modern day tomato including lycopene beta cyclase that mediates conversion of lycopene to *β*-carotene. The fruit specific *β*-carotene formation is mediated by a chromoplast-specific paralog of lycopene beta cyclase (*CYC-B)* gene. Presently limited information is available about how the variations in *CYC-B* gene contributed to its neofunctionalization. *CYC-B* gene in tomato clade contained several SNPs and In-Dels in the coding sequence (33 haplotypes) and promoter region (44 haplotypes). The *CYC-B* gene coding sequence in tomato appeared to undergo purifying selection. The transit peptide sequence of CYC-B protein was predicted to have a stronger plastid targeting signal than its chloroplast specific paralog indicating a possible neofunctionalization. In promoter of two *B*^*og*^ (*Beta old gold*) mutants, a NUPT (nuclear plastid) DNA fragment of 256 bp, likely derived from a *S*. *chilense* accession, was present. In transient expression assay, this promoter was more efficient than the “*Beta* type” promoter. CARGATCONSENSUS box sequences are required for the binding of the MADS-box regulatory protein RIPENING INHIBITOR (RIN). The loss of CARGATCONSENSUS box sequence from *CYC-B* promoter in tomato may be related to attenuation of its efficiency to promote higher accumulation of *β*-carotene than lycopene during fruit ripening.

## Introduction

Tomato is one of the commonly consumed vegetables and is also a principal dietary source of several antioxidants, such as carotenoids, tocopherols and flavonoids. The major antioxidant present in ripe tomato fruit are carotenoids. During ripening the chloroplasts are transformed to chromoplasts with upregulation of carotenoid biosynthesis along with progressive degreening of tomato fruits [1]. Ripe tomato fruits mainly accumulate red coloured lycopene followed by *β*-carotene and also have little amounts of other carotenoids such as violaxanthin, lutein, phytoene, phytofluene, and neurosporene [2, 3].

The carotenoid biosynthesis pathway in ripening tomato fruit is essentially similar to that operates in photosynthetic tissue such as leaf, with few fruit specific modification during chloroplast to chromoplast transition. One of the modifications is the recruitment of two chromoplast-specific enzymes catalyzing conversion of geranyl-geranyl pyrophosphate to phytoene and lycopene to *β*-carotene. In higher plants a chloroplast-specific lycopene *β*-cyclase enzyme (LCYB) mediates the conversion of lycopene to *β*-carotene in photosynthetic tissue [4]. The lycopene to *β*-carotene conversion in chromoplast is mediated by a paralog of lycopene *β*-cyclase which is called as ‘chromoplast-specific *lycopene beta cyclase’* gene (*CYC-B*) in tomato. While CYC-B enzyme retains its catalytic function similar to LCYB, it has only ca.55% amino acid sequence identity with LCYB1 and LCYB2. *CYC-B* is an intronless gene with a total size of 1.6 Kb (including the UTR region), coding for a protein of 498 amino acids [4].

In most tomato varieties the lycopene level in fruits is, at least, ten times higher than *β*-carotene. There have been concerted efforts to alter the lycopene levels in tomato by introgression of genes from its wild relatives and by mutations. The loss of function mutation in *CYC-B* gene can lead to increase in lycopene level, whereas gain of function can lead to high *β*-carotene level. The *B* allele (*CYC-B* allele present in *Beta* mutant) was introduced into the cultivated tomato during crossings with its wild relative *Solanum habrochaites* for obtaining tomato lines with high *β*-carotene levels. It was later established that *CYC-B* expression increases dramatically during the breaker stage of ripening in ‘*Beta’* mutant leading to higher accumulation of *β*-carotene [4]. Null mutations in the *CYC-B* gene stimulate lycopene accumulation in fruits of *old-gold* (*B*^*og*^) [5, 6] and *old-gold-crimson* (*og*^*c*^) mutants of tomato [4]. A loss of function mutation *in CYC-B* gene (A949G) increases lycopene and total carotenoid content of ripe tomato fruit [7].

Currently, wild and cultivated tomatoes belonging to tomato clade are classified into 4 groups [8]: Lycopersicon group [*S*. *lycopersicum*, *S*. *pimpinellifolium*, *S*. *cheesmaniae* and *S*. *galapagense*], Neolycopersicon group (*S*. *pennellii*), Eriopersicon group [*S*. *peruvianum*, *S*. *corneliomulleri*, *S*. *huaylasense*, *S*. *habrochaites*, and *S*. *chilense*], and Arcanum group [*S*. *arcanum*, *S*. *chmielewskii*]. The high accumulation of lycopene with red fruits is a recent evolutionary event and is present only in two members of Lycopersicon group viz. *S*. *lycopersicum* and *S*. *pimpinellifolium*; two other members *S*. *cheesmaniae* and *S*. *galapagense* have yellow/orange fruits. The other members of tomato clade belonging to Neolycopersicon, Eriopersicon and Arcanum groups have green fruits. The evolutionary steps involved in the transformation of green fruits to red fruits within tomato clade are being investigated using several approaches including comparative genomics [9–11].

The variation in fruit coloration in members of tomato clade may represent differences in the expression patterns of genes regulating carotenoid biosynthesis pathway. In addition, the changes in the coding sequence of above genes might also alter the gene function. In tomato, it has been suggested that genome triplication in Solanum ancestor followed by neofunctionalization of genes have aided to the evolution of red coloured fleshy fruit [12]. Among these genes, the *CYC-B* gene, a paralog of lycopene *β*-cyclase, also underwent neofunctionalization for fruit chromoplast-specific expression. The comparison of CYC-B homologues among different plant species and *CYC-B* gene/promoter sequence in the members of tomato clade thus has the potential to decipher chromoplast- specific regulation of *β*-carotene accumulation in tomato fruits.

In the present study, we examined genetic variations (SNPs, Insertions and Deletions) in *CYC-B* gene and its promoter in various tomato accessions using EcoTILLING. The *CYC-B* variants from data deposited in ‘tomato variant browser’ were also analyzed along with the probable effect of variations on gene function. *In-silico* analysis of the *CYC-B* promoter region was carried out to identify the probable response elements. The relative efficiencies of three promoter sequences representative of variable *CYC-B* promoters were compared by transient expression assay. We report that in tomato clade the *CYC-B* gene coding sequence appears to undergo purifying selection. The higher variations in promoter region than in the coding region of *CYC-B* gene is likely related to regulatory neofunctionalization (R-NF) of *CYC-B* gene in tomato clade. The neofunctionalization of *CYC-B* gene was also aided by diversification of plastid transit peptide sequence. Our results suggest that neofunctionalization of genes may have played a larger role in tomato clade for fruit colour development.

## Materials and Methods

### Plant material

A population consisting of 484 accessions belonging to tomato and its wild relatives was examined for SNPs by EcoTILLING. The accessions were obtained from NBPGR (New Delhi, India), IIVR (Varanasi, India), TGRC (California, USA) and Bejo Sheetal Seeds (Jalna, India) (“S1 File”). *S*. *lycopersicum cv*. Arka Vikas (originally obtained from Indian Institute of Horticulture Research, Bangalore) was used as the reference variety. The wild tomato species used in the study are *S*. *pimpinellifolium* (LA1589), *S*. *cheesmaniae* (LA0483), *S*. *habrochaites f*. *glabratum* (LA1362), *S*. *neorickii* (LA2133), *S*. *pennellii* (LA0716) and *S*. *chilense* (LA0458). DNA extraction from each accession was carried out by the protocol outlined by Sreelakshmi et al. [13].

### Detection of promoter size variations

The *CYC-B* promoter was amplified from various accessions using the forward and reverse primers 5′-CAATCCAGTGGATTCTCGTTCTGGCACCT-3′ and 5′-TATTGATGCTTTTGGGTACCTACTTTGGC-3′ respectively designed to amplify 1007 bp fragment (reference cultivar) with an overlap in the exon region.

### SNP detection using EcoTILLING

Screening for SNPs was done by a protocol described by Till et al [14, 15] and Colbert et al [14, 15] with few modifications. The sequence of *CYC-B* gene (Solyc06g074240.1) and ca. 1 Kb upstream region was obtained from SGN database [16]. Nested PCR approach was used with primers for the first step PCR designed to cover both the coding region and ~1 Kb upstream sequence of the coding region with an expected amplicon size of 2684 bp. The primers for the second step PCR (with Universal M13 tails) were designed to amplify 991 bp and 1231 bp fragments of the upstream sequence and the coding sequence respectively using, first step PCR product (Primers details are in “S2 File”).

PCRs were set up in 96-well microtiter plates with 20 μL reaction volume. *Step I*: Pre-amplification mixing of the genomic DNA samples of each accession was done with that of Arka Vikas in a 1:1 ratio and 5 ng of template was added to each reaction. Other components included 1X PCR buffer, 0.2 mM each dNTPs, 0.18 μL Taq polymerase (in-house isolated) and 0.15 pmoles each of forward and reverse primers. The thermocycling conditions for amplification were 94°C-4 min; 10 cycles of 94°C-20 sec, 67°C-45 sec, 72°C-2 min 30 sec; step down: 25 cycles of 94°C-20 sec, 60°C-45 sec, 72°C-2 min 30 sec; 72°C-10 min; held at 4°C. *Step II*: Appropriately diluted I step PCR products were used as the template for the second step PCR. 0.10 μL Taq polymerase and 0.3 pmoles of primer cocktail in the ratio 2:3:1:4 (Forward unlabelled: M13-Forward labeled: Reverse unlabelled: M13-Reverse labeled) was used. The thermocycling conditions for amplification were 94°C-4 min; 10 cycles of 94°C-20 sec, 67°C-45 sec, 72°C-1 min 20 sec; step down: 25 cycles of 94°C-20 sec, 60°C-45 sec, 72°C-1 min 20 sec; 72°C-10 min; heteroduplex formation: 99°C-10 min, 80°C-20 sec, 70 cycles of 80°C-7 sec with a decrement of 0.3°C per cycle and held at 4°C. The mismatch cleavage reaction and detection was performed using the protocol described by Sreelakshmi et al. [13] with 20 μL PCR product in a total volume of 45 μL.

### Sequencing and analysis of sequences

All sequencing reactions were carried out by Sanger dideoxynucleotide chain termination method (Bioserve Biotechnologies (India) Pvt. Ltd). Sequences were assembled using Chromas software. The zygosity of the SNPs was determined based on the presence of single (homozygous) or double (heterozygous) peaks in the chromatogram. The amino acid changes corresponding to each nucleotide changes and change in restriction enzyme sites were predicted using PARSENP (Project Aligned Related Sequences and Evaluate SNPs) analysis [17]. The probable effects of the nonsynonymous changes on protein functions were predicted using SIFT (Sorting Intolerent From Tolerent) software [18]. Phylograms were constructed using MEGA6 [19]. The evolutionary history was inferred using the Neighbor-Joining method [20]. Transition/transversion bias (R) was calculated using MEGA6 [19]. Substitution pattern and rates were estimated under the [21] 2-parameter model. The haplotypes were identified using DnaSP software [22]. The promoter sequences were analyzed using PLACE (PLAnt Cis-acting regulatory DNA Elements) software to identify the regulatory elements [23]. Comparative alignment diagram for the promoter sequences was generated using PlantPAN [24]. Subcellular localization scores were predicted using *‘TargetP 1*.*1’* software [25].

### Transient expression

*Constructs*: The promoter fragments were amplified using the primer sequences 5′- ACACCAGGGTTGTCAAAAATGTCTC - 3′ and 5′- TATAGAGAATGTATAAGATTGATAATGGT- 3′ and cloned into the double luciferase vector pGreenII 0800-LUC upstream to the *FLUC* (Firefly luciferase) gene [26]. *Fruit tissues*: Fruit tissues of tomato cv. Arka Vikas at 30 DPA stage was used for transient expressions experiments. The fruits were washed and surface sterilized with 75% (v/v) ethanol. Thin slices of mesocarp fruit tissues (3–5 mm Χ 3–5 mm) were arranged on a sterile filter paper (3 cm diameter) at the center of petriplates (9 cm diameter) with an osmotic medium (0.4 M mannitol, 0.4 M sorbitol, 0.8% agar, 0.1 M sucrose). The plates were sealed with parafilm and incubated for 4 hrs at 26°C. *Tungsten-DNA mixture*: 1 μg of the plasmid DNA sample of each construct was mixed with 50 μL aliquots of 1.1 μ size sterile tungsten particle (Bio-Rad Laboratories, Inc., Hercules, CA) suspended in sterile distilled water (SDW) at a concentration of 50 mg/ml. To this 50 μL of 2.5 M CaCl_2_ and 20 μL of 100 mM spermidine were added. The tubes were vortexed briefly and placed on ice for 5 min. When the DNA-coated tungsten particles settled down, around 50 μL of the clear supernatant was discarded, and the rest of the aliquot was used for microprojectile bombardment. *Transformation of fruit tissues*: Each construct was transformed to the fruit tissues in three replicates using Particle Inflow Gun as described by Finer et al. [27]. The bombarded tissues were then left on the osmotic medium (petriplates sealed with parafilm) and incubated at 26°C and 45% relative humidity with 16 h light / 8 h dark photoperiod for 48 hrs for post-bombardment treatment.

### RNA extraction and Real-time PCR

Fruit tissues transformed with each construct were frozen in liquid N_2_. and total RNA was extracted using TRI Reagent (Sigma, St. Louis, MO, USA), according to the manufacturer’s protocol. To remove the contaminating DNA, all the RNA samples were treated with DNase, using RQ1 RNase-Free DNase kit (Promega, Madison, WI, USA) according to manufactures protocol. Reverse transcription was performed with 2 μg of total RNA in a total volume of 20 μL using SuperScript^™^ III first-strand synthesis system for RT-PCR (Invitrogen, Rockville, MD, USA) following manufactures protocol. Real-Time PCR was performed using cDNAs corresponding to 5 ng of total RNA in 10 μL reaction volumes with 0.2 pmoles each of forward and reverse primers using the SYBR Green PCR Master Mix (Takara, Otsu, Japan) on a 7300 Fast Real-Time PCR system (Applied Biosystems, Foster City, CA, USA). *FLUC* expression in the empty vector pGreenII 0800-LUC was used to normalize any background level expression. Expression of *β-actin* was used for normalization between samples. The primer sequences used for Real-Time PCR designed using primer3 software are *FLUC*F: 5′- GAGGCGAACTGTGTGTGAGA - 3′, *FLUC*R: 5′- GTGTTCGTCTTCGTCCCAGT - 3′, *β-actin*F: 5′-GAAATAGCATAAGATGGC - 3′, *β-actin*R: 5′-ATACCCACCATCACACCA - 3′. Relative *FLUC* expression under *Beta* (*B*) type and LA0348 type promoters were calculated with respect to the *FLUC* expression under WT promoter using the formula 2^(-ΔΔCT)^ (ΔCt = Ct value of *FLUC*—Ct value of *β-actin*).

### Dual Luciferase assay

The quantity of Firefly luciferase and *Renilla* luciferase were assayed using the dual luciferase assay reagents (Promega, Madison, WI, USA) following manufactures instructions. Chemiluminescence measurements were made using GloMax^®^ 96 Luminometer (Promega, Madison, WI, USA).

### Variant data resource

SNPs and In-Dels annotated for landraces, heirloom cultivars and wild relatives were obtained from the ‘tomato 100+ variant browser' [28]. List of accessions used for comparison and analysis of genetic variations is given in “S3 File”.

## Results

### Genetic variations in *CYC-B* gene and promoter

**Fig 1:**
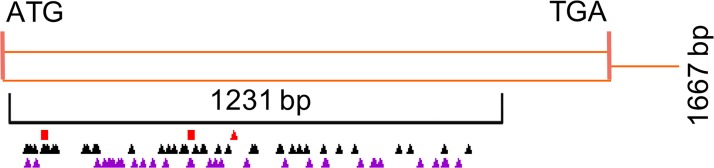
Distribution of SNPs and In-dels in *CYC-B* gene. Variants identified using Eco-TILLING and Sanger sequencing, and from tomato structural variant browser are represented with a modified PARSESNP output diagram. Black and purple triangles indicate the positions of nonsynonymous and synonymous nucleotide substitutions respectively. Red triangle represents the position of the nonsense substitution G570A in tomato accession EC20636. The red squares represent frameshift changes.

The genetic variations in *CYC-B* gene (Solyc06g074240.1.1) and its promoter region in 484 tomato accessions including few wild relatives and two *CYC-B* mutants; *Beta* (*B*) (LA3000) and *Beta old gold* (*B*^*og*^) (LA0348) were compared with tomato cultivar Arka Vikas (Sel 22). The examined region included ca. 74% (1231 bp) of the coding region and 876 bp (−1 to −876) of the promoter of *CYC-B* gene. Position and nature of polymorphism in the coding region of *CYC-B* gene are described in “Fig 1” and “S4 File”. The extent of the polymorphism appears to be related to evolutionary divergence, as maximum number of SNPs were observed in wild relative *S*. *pennellii* (16) followed by *S*. *chilense* and *S*. *habrochaites* (13 each), *S*. *neorickii* (12), *S*. *cheesmaniae* (2) and the minimum in *S*. *pimpinellifolium* (1). Out of 50 different SNPs, 18 were common in wild relative(s) and other accession(s). The classification of SNPs after excluding wild relatives revealed 8 haplotypes in the coding region and 9 haplotypes in the promoter region. The *CYC-B* promoter in tomato accessions was highly polymorphic compared to the coding region as evident by 5 time higher SNP frequency (7.73 x 10^−4^) than the coding region (1.58 x 10^−4^).

The presence of extra sequence elements in the promoter region of *B* allele compared to *b* (wild type) reported by Ronen et al. [4] prompted us to examine the amplicon size for the promoter region in all accessions. Similar to *Beta* mutant (~50 bp), the green-fruited wild relatives and few tomato accessions had a bigger amplicons size compared to Arka Vikas indicating the presence of extra sequence(s) in *CYC-B* promoter. The presence of extra sequence was restricted to the promoter, and such insertion in ORF was not observed in any other tomato accession. Apparently the promoter region of *CYC-B* is hypervariable compared to the coding region.

In addition to examining SNPs in 484 tomato accessions, we also used tomato variant browser to identify genetic polymorphism in ORF (1231 bp) of *CYC-B* gene and its promoter (−1 to −876 bp) with reference to Arka Vikas cultivar [9]. While the EcoTILLING identified 19 tomato accessions, the usage of variant browser identified additional 37 accessions with the polymorphism in *CYC-B* gene, predominantly in tomato wild relatives (S4 File). Out of 83 variant sites identified, 28 were common in both sets, 33 were detected using the variant browser, and 22 were obtained from EcoTILLING. Highest SNP frequency was observed in Eriopersicon group, followed by Arcanum, Neolycopersicon and Lycopersicon groups.

Interestingly examination of *CYC-B* gene in all tomato accessions revealed no insertion or deletion other than already reported frame shift mutations (A103: and A463ATA). The SIFT analysis predicted 9 nucleotide substitutions from various accessions to affect the CYC-B protein function (“S5 File”). Based on the distribution of SNPs in *CYC-B* gene, 33 haplotypes are identified from 588 accessions (“S6 File”). Distribution of various synonymous, nonsynonymous, nonsense substitutions and In-dels in *CYC-B* gene of various haplotypes are represented with PARSESNP output diagram in “S7 File” and restriction enzyme polymorphisms changes are shown in “S8 File”. The highest number of haplotypes (16) were in Eriopersicon group followed by Lycopersicon and Arcanum groups (6 haplotypes each) and Neolycopersicon group (2 haplotypes). Both haplotype-and group-specific SNPs were identified. Arcanum group had three specific SNPs (T125C, G402A, A830T), whereas G390T was Eriopersicon specific. Ten SNPs were exclusively present in green fruited species. Three SNPs (T325C, T369C, G1029A) were specific to *S*. *habrochaites* and one SNP (A108T) was specific to *S*. *huaylense*. The haplotype 24 and haplotype 32 had several SNPs different from all other accessions. In almost all accessions, nonsynonymous nucleotide change G868A (D290N) was consistently present indicating it to be a recent nucleotide change.

**Fig 2:**
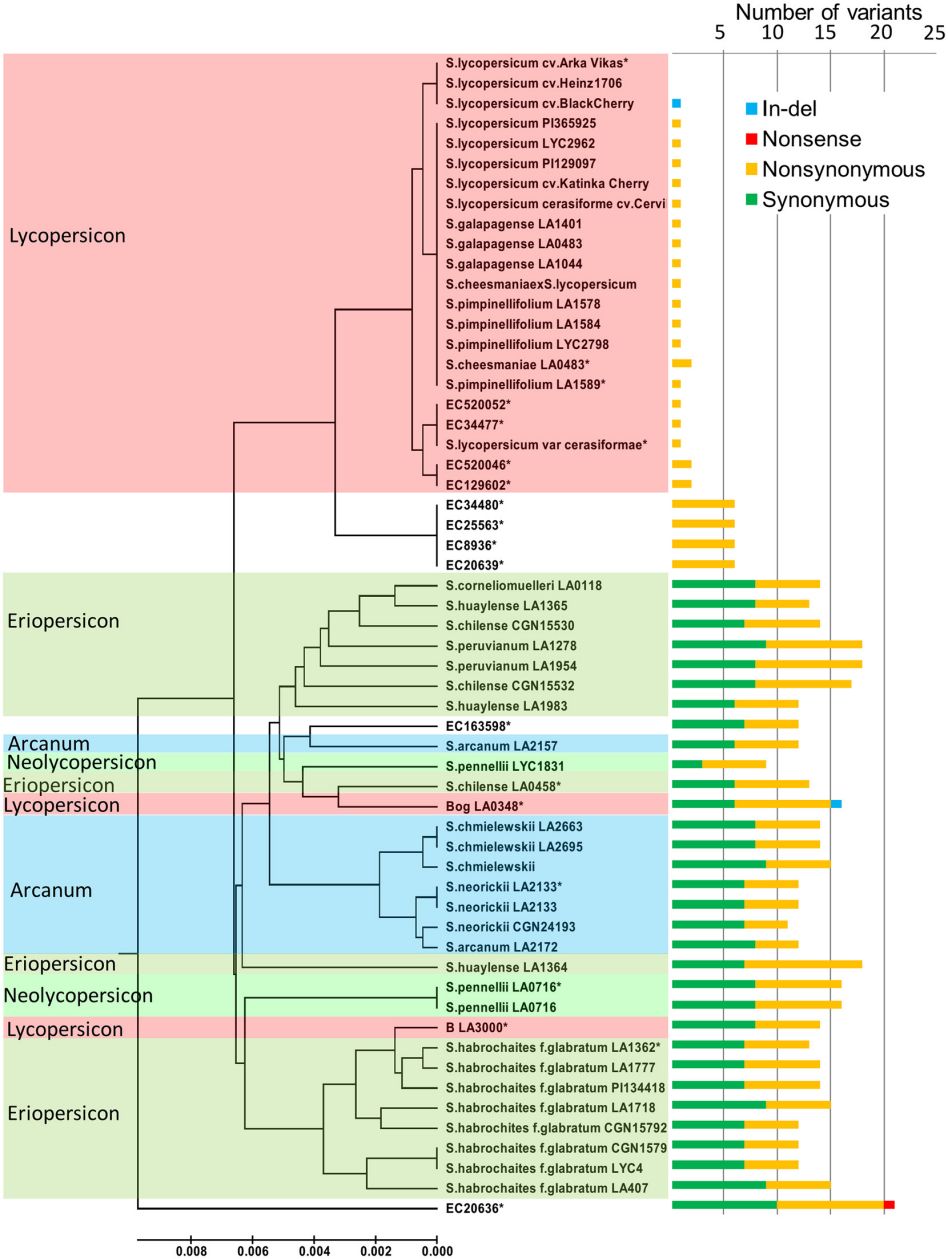
Evolutionary relationships of *CYC-B* gene sequences from 58 tomato accessions. The evolutionary history was inferred for 1098 bp region of the gene using MEGA6. The optimal tree with the sum of branch length = 0.11 is shown. The tree is drawn to scale, with branch lengths in the same units as those of the evolutionary distances used to infer the phylogenetic tree. Number of variants present in *CYC-B* gene for each accession is represented with bars (right).

Phylogenic tree constructed using sequences from 58 accessions indicates the evolutionary pattern of the gene in these accessions. The accession LA2157 (*S*. *arcanum*) did not cluster with Arcanum group but clustered with EC163598. Interestingly one accession of *S*. *pennellii* (LYC1831) appears to be more close to the Lycopersicon group. A sole exception was EC20636 which did not cluster with any group as it had several unique SNPs (“Fig 2”).

In addition to SNPs, *CYC-B* promoter also had several In-dels with more insertions (1 to 27 bp) than deletions (1 to 4 bp). The presence of the sequence ‘GAACCCAAACTC’ (upstream to −752 of Arka Vikas) in most of the accessions including three tomato accessions (Katinka Cherry, PI 129097, LYC 2962) suggests a very recent deletion event in *CYC-B* promoter. One deletion each was present in accessions Katinka Cherry and PI 129097. While the EcoTILLING identified 13 tomato accessions, the usage of variant browser identified additional 34 accessions with variants. The accessions were classified into 44 haplotypes, with 532 accessions in Haplotype 1 along with the reference cultivars. The Lycopersicon group has an equal number of haplotypes as Eriopersicon (16 each), Arcanum has 6 haplotypes, and Neolycopersicon has 3 very diverse haplotypes (“S9 File”).

**Fig 3:**
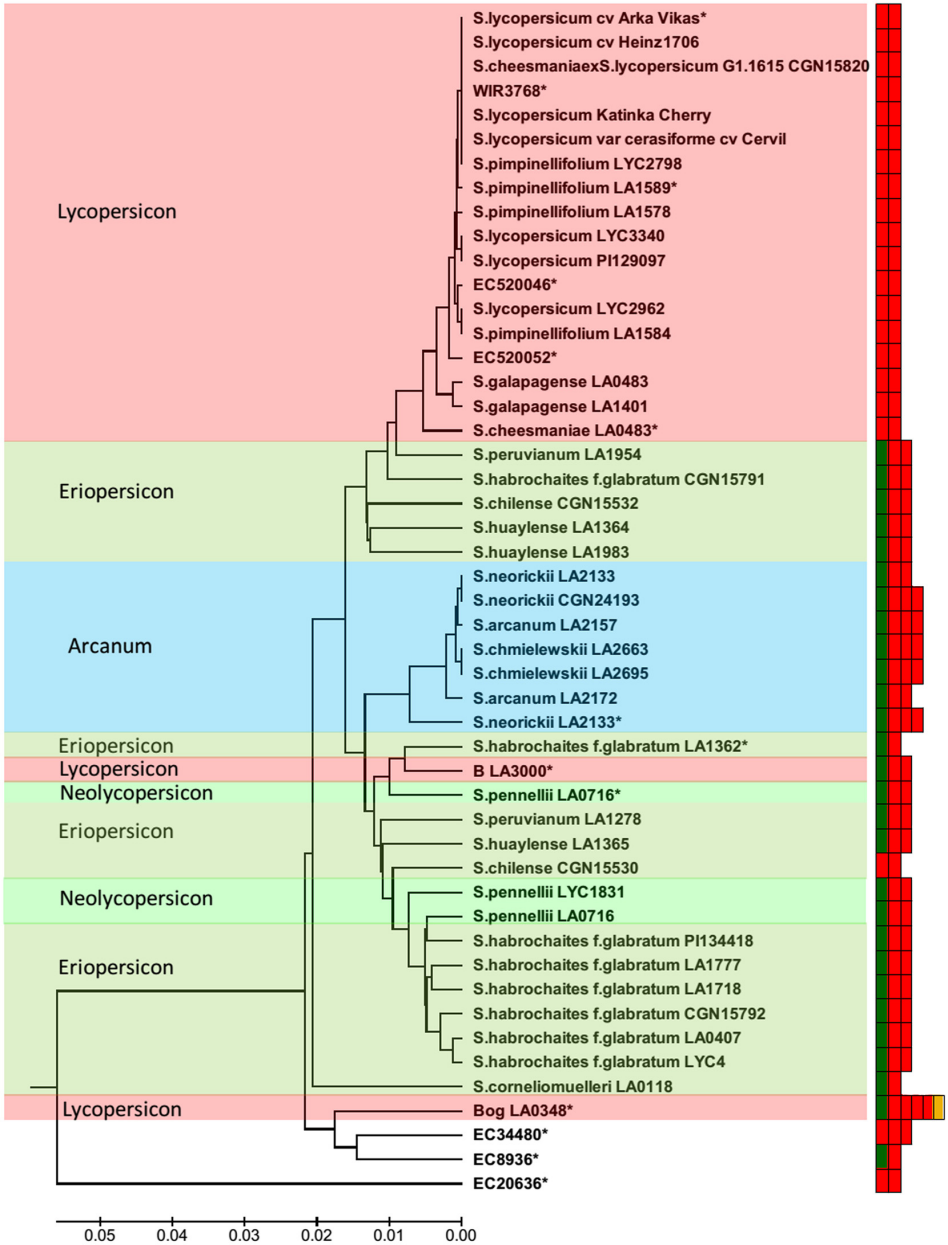
Evolutionary relationships of *CYC-B* promoter sequences from 49 tomato accessions. All sequences corresponding to –1 to –876 bp region of the reference variety (*S*. *lycopersicum* cv. Arka Vikas) was used for constructing the tree using MEGA6. The optimal tree with the sum of branch length = 0.38 is shown. The number of RIN binding sites in the *CYC-B* promoter sequence of the accessions are indicated with coloured boxes (right): CARGATCONSENSUS (green), CARGCW8GAT (red) and CARGNCAT (yellow).

Phylogenic tree constructed using 49 *CYC-B* promoter sequences showed a different grouping of the accessions compared to ORF. Along with Lycopersicon group, five accessions of the Eriopersicon group were also clustered, while remaining accessions of Eriopersicon group clustered with Arcanum and Neolycopersicon group. Interestingly the accessions of Neolycopersicon grouped to different subclusters. The *CYC-B* promoter of *Beta* mutant (LA3000) was highly similar to LA1362 (*S*. *habrochaites*) and LA0716 (*S*. *pennellii*) indicating its origin from wild relatives [4]. Three green fruited accessions (EC34480, EC8936 and EC20636) were found to be the most primitive accessions with respect to the promoter sequences. For promoter too, EC20636 is highly divergent than all other wild relatives (“Fig 3”).

### *CYC-B* gene undergoes purifying selection

The overall nucleotide diversity (π) in the *CYC-B* promoter sequences (0.0296) was 2 fold higher than coding sequences (0.0105). In green fruited species, it was 0.0319 in promoter and 0.0127 in exon, whereas, in coloured fruited species, the nucleotide diversity of promoter sequences (0.0121) was ~10 fold higher than for coding region (0.0018). Tajima’s D for overall promoter sequences was negatively significant (−1.8601, P < 0.05) indicating the presence of rare alleles. However, considering only coloured fruited species, Tajima’s D for promoter sequences is non-significant (−1.7077, 0.10 > P > 0.05) indicating neutral evolution of *CYC-B* promoter sequences in coloured fruited species. Even though for the exon region of coloured fruited species, Tajima’s D is negatively significant (−1.93, P < 0.05), Tajima's D (NonSyn) is non-significant (-1.28012, P > 0.10). In general, Tajima’s D is non-significant for the exon region (-1.19577, P > 0.10), indicating a neutral evolution. The Ka/Ks values for all accessions and the coloured-fruited accessions are 0.186 and 0.752 respectively. It clearly shows purifying selection of *CYC-B* gene.

### *CYC-B* gene in colored fruited species has more transversions than transitions

**Fig 4:**
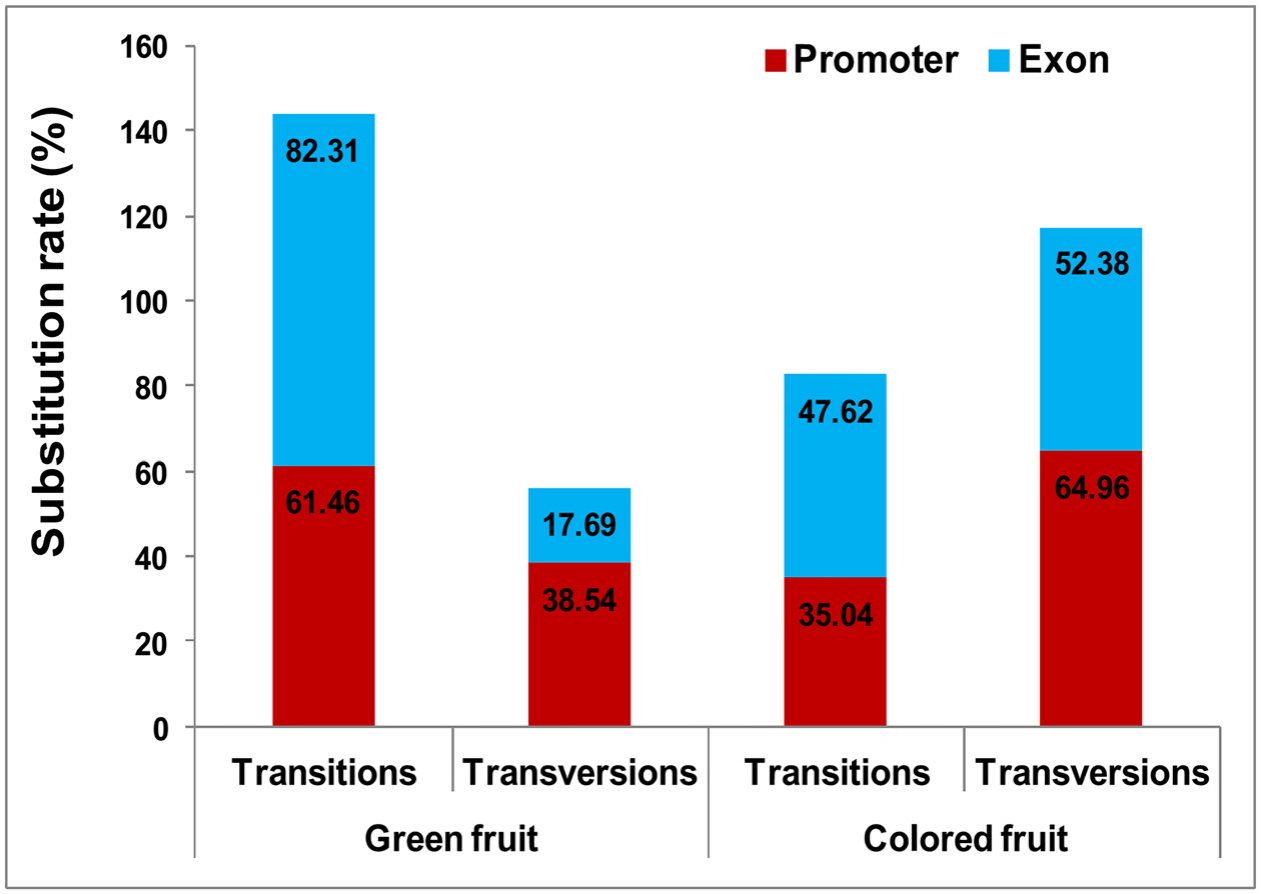
Frequency of transition/transversion ratios in *CYC-B* gene and promoter. Green fruited accessions have more transitions, whereas, coloured fruited accessions carry more transversions.

In general, the *CYC-B* coding region had more transitions (transition/transversion ratio, kappa = 1.85), whereas, promoter region had more transversions (kappa = 0.93). The overall transition/transversion bias (R) for coding and promoter sequences were 3.33 and 1.61 respectively. Nevertheless, the coding sequence in colored fruited species had comparatively high transversion rate (kappa = 0.91). Green fruited species have a marginally higher rate of transition than transversions for coding and promoter sequences (kappa = 2.56), whereas, in colored fruited species transversions dominate (kappa = 0.70). Interestingly, the transversion rate in the promoter sequence of coloured fruited species is almost double the rate of transitions (kappa = 0.54) (“Fig 4”).

### EC20636 harbours a novel loss of function allele of *CYC-B*

Currently in tomato, only two loss of function mutations are known for *CYC-B* namely, *Beta old gold* (*B*^*og*^ A463ATA) and *Beta old gold crimson* (*B*^*c*^ A103:)[4]. Analysis of polymorphism in *CYC-B* gene in this study identified two new deleterious SNPs in EC20636. The first SNP (A317C) changed lysine to threonine at position 106 in CYC-B while second SNP (G570A) inserted a stop codon TGA leading to truncated CYC-B protein with 189 amino acids (“Fig 1”). However, EC20636 is a green-fruited accession that accumulates little carotenoid in fruits, therefore, influence of this SNP on fruit phenotype was not discernible. Nonetheless, above loss of function *CYC-B* allele potentially can be used for introgression into commercial cultivars for developing a high lycopene tomato fruit.

### A NUPT (nuclear plastid) DNA fragment appears to increase *CYC-B* promoter efficiency

**Fig 5:**
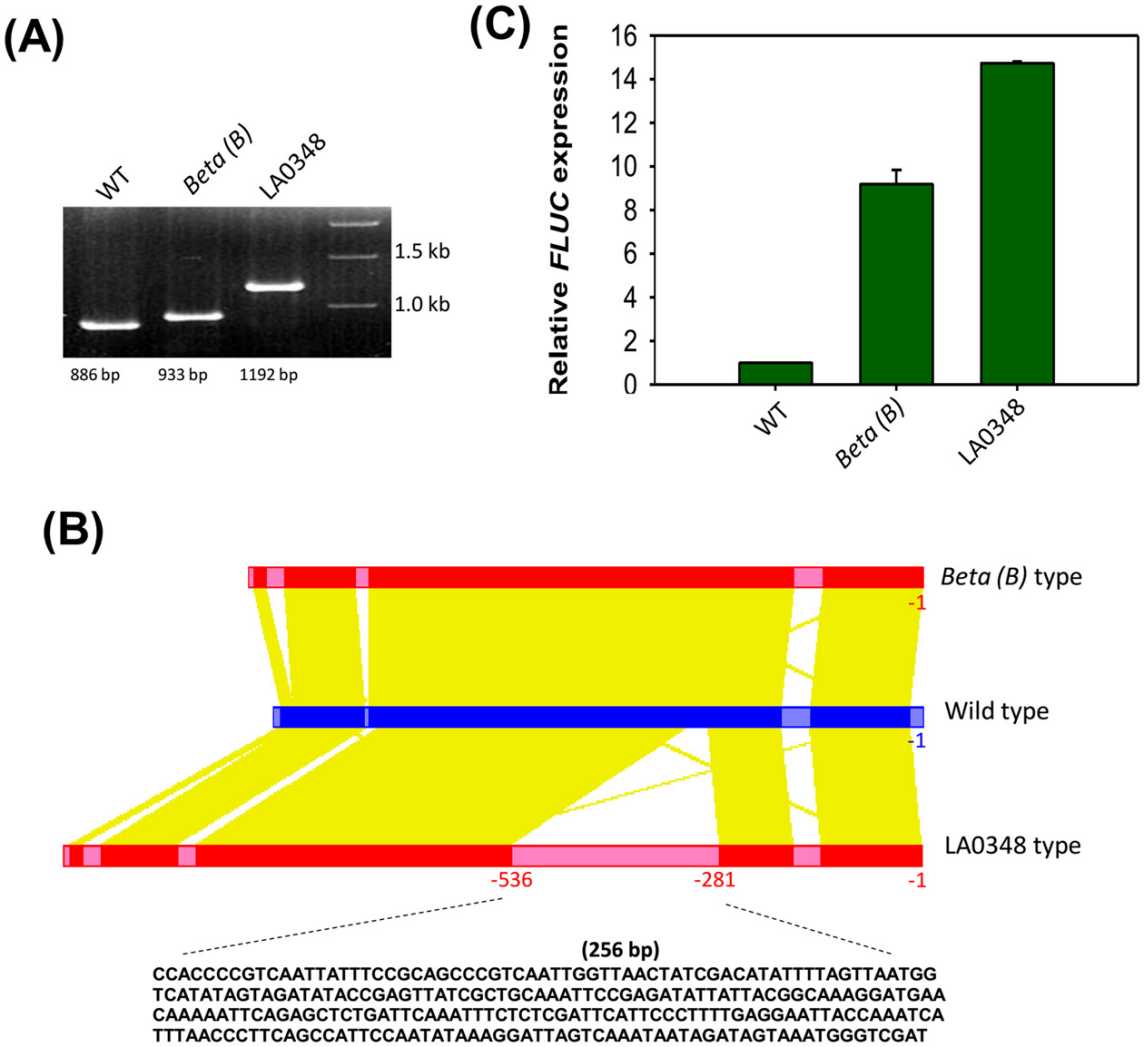
Comparison of three different *CYC-B* promoter sequences. *CYC-B* promoter fragments amplified from WT (Arka Vikas), *Beta (B)* mutant (LA3000) and *B*^*og*^ mutant (LA0348) used for transient expression **(A)**. Modified diagram generated using PlantPAN shows the alignment of the promoter sequences and 256 bp insertion in *B*^*og*^ (LA0348) **(B)**. Relative expression of *FLUC* under different promoters in pGreenII 0800-LUC constructs **(C)**.

PCR amplification of *CYC-B* promoter revealed three tomato accessions (LA0348 and, LA0500-accessions with *B*^*og*^ mutation, LA0458-a *S*. *chilense* accession) with promoter larger than Arka Vikas (“Fig 5A”). The sequencing of promoter from these three accessions showed four insertions that were identical to *Beta* (*B*) mutant promoter and an additional 256 bp insertion upstream to −281 bp (“Fig 5B”). Above 256 bp insertion was examined for sequence similarity with other regions on tomato genome(s). The BLAST search in SGN database showed similarity to tomato chloroplast genome sequence [AM087200.3 (1e-105)] and *Petunia axillaris* ribosomal protein S4 (rps4) gene of chloroplast [HQ385116.1 (7e-94)]. Probably, this insertion arose due to integration of a fragment of chloroplast DNA in the *CYC-B* promoter. Since an identical insertion is present in *S*. *chilense*, above insertion in tomato was likely mobilized during breeding of *B*^*og*^ line. The *B*^*og*^ mutant line was obtained after crossing *S*. *chilense* and *S*. *lycopersicum* [5] and ‘*old gold’* phenotype was transferred to *S*. *lycopersicum* by nine successive backcrosses [6]. The existence of the fragment in two accessions of *B*^*og*^ and in one accession of *S*. *chilense* indicates the probability of introgression of the fragment from *S*. *chilense* during crossing.

It is reported that in fruits of ‘*Beta’* mutant, high *β*-carotene content results from more efficient ‘*Beta* type’ promoter than the normal *CYC-B* promoter [4]. In this study, an additional promoter ‘LA0348 type’ was identified from two accessions bearing *B*^*og*^ mutation and one accession of *S*. *chilense*. However, *B*^*og*^ mutants do not accumulate *β*-carotene and in *S*. *chilense* the carotenoid biosynthesis pathway is not upregulated during fruit ripening, thus precluding evaluation of ‘LA0348 type’ promoter activity in these accessions. Consequently, the efficiency of different promoters was evaluated by transient expression using promoter-reporter constructs. Three highly different promoter sequences, representative of the various promoters identified, were selected for the transient expression assay. The promoter efficiency was evaluated by levels of luciferase expression driven by ‘Wild-type’ (Arka Vikas), ‘*Beta* type’ (*Beta*, LA3000) and ‘LA0348 type’ (*B*^*og*^, LA0348) promoters. Since in transient expressions, the level of luminescence was weak, the promoter efficiencies were compared by measuring the relative *FLUC* transcript abundance. Interestingly ‘*Beta* type’ promoter showed ~9-fold higher *FLUC* expression compared to ‘Wild type’ promoter, whereas, enhancement was ~15-fold with ‘LA0348 type’ promoter (“Fig 5C”). Apparently the additional insertion in ‘*Beta* type’ promoter leads to further enhancement in its expression efficiency.

### *CYC-B* promoter efficiency appears to be related to number of RIN binding sites

The analysis of *CYC-B* promoter sequences using PLACE (PLAnt Cis-acting regulatory DNA Elements) software showed the presence of a number of *cis*-elements varying in the type and number of repetitions. One interesting difference was in the ‘CArG’ box binding sites of transcription factor-RIN, which is considered as a master regulator of ripening [29, 30]. In *CYC-B* promoter sequences three different ‘CArG’ boxes were identified (“Table 1”): CARGATCONSENSUS, CARGCW8GAT and CARGNCAT. Among these CARGCW8GAT was present in all the accessions and was the only type of ‘CArG’ box present in Lycopersicon group (“Fig 3”). Based on the type and number of ‘CArG’ boxes, the accessions were classified into 6 types. *Type I*: Accessions with 2 CARGCW8GAT boxes. It included all Lycopersicon group members, *S*. *chilense* (CGN15530) and EC20636 with a highly divergent *CYC-B* sequence. *Type II*: Consisted of only one accession (EC34480) with 3 CARGCW8GAT boxes. *Type III*: Consisted of three accessions with 1 CARGCW8GAT box and 1 CARGATCONSENSUS. *Type IV*: Most Eriopersicon group members, all Neolycopersicon accessions and mutant LA3000 belonged to this type and characterized by the presence of 2 CARGCW8GAT and 1 CARGATCONSENSUS boxes. *Type V*: Most Arcanum group members belonged to this group with 3 CARGCW8GAT boxes and 1 CARGATCONSENSUS. *Type VI*: The *B*^*og*^ mutant accession (LA0348) with 4 CARGCW8GAT, 1 CARGATCONSENSUS and 1 CARGNCAT box belonged to this group (“Fig 3”, “Table 1”).

**Table 1:** List of RIN binding sites identified in the *CYC-B* promoter sequences of various accessions using PLACE Signal Scan Search.

Type	Accession	Factor or Site Name^@^	Locus
I	*S*. *lycopersicum* (cv Arka Vikas*, cv. Heinz 1706, LYC2962, LYC3340, *Cerasiforme* cv. Cervil), *S*. *cheesmaniae* (LA0483*), *S*. *cheesmaniae x S*. *lycopersicum* G1.1615 (CGN 15820), *S*. *pimpinellifolium* (LA1584, WIR3768*, LA1578, LYC2798), *S*. *galapagense* (LA0483, LA1401), *S*. *chilense* (CGN15530)	CARGCW8GAT	−98, −140
	*S*. *lycopersicum* (Katinka Cherry, PI129097), *S*. *pimpinellifolium* (LA1589*), EC520052*, EC520046*	CARGCW8GAT	−97, −139
	EC20636*	CARGCW8GAT	−102, −398
II	EC34480*	CARGCW8GAT	−102, −144, −695
III	*S*. *habrochaites f*. *glabratum* (LA1362*)	CARGATCONSENSUS	−632
		CARGCW8GAT	−97
	EC8936*	CARGATCONSENSUS	−646
		CARGCW8GAT	−102
	*S*. *corneliomuelleri* (LA0118)	CARGATCONSENSUS	−638
		CARGCW8GAT	−96
IV	*S*. *pennellii* (LA0716)*	CARGATCONSENSUS	−639
		CARGCW8GAT	−98, −140
	*B* (LA3000*)	CARGATCONSENSUS	−637
		CARGCW8GAT	−97, −139
	*S*. *neorickii* (LA2133*)	CARGATCONSENSUS	−638
		CARGCW8GAT	−98, −655
	*S*. *habrochaites f*. *glabratum* (CGN15791)	CARGATCONSENSUS	−630
		CARGCW8GAT	−98, −140
	*S*. *habrochaites f*. *glabratum* (PI134418)	CARGATCONSENSUS	−627
		CARGCW8GAT	−98, −140
	*S*. *huaylense* (LA1364)	CARGATCONSENSUS	−643
		CARGCW8GAT	−101, −692
	*S*. *peruvianum* (LA1954)	CARGATCONSENSUS	−624
		CARGCW8GAT	−98, −140
	*S*. *chilense* (CGN15532), *S*. *peruvianum* (LA1278), *S*. *habrochaites f*. *glabratum* (CGN15792, LA1718, LA1777, LA040, LYC4), *S*. *pennellii* (LYC1831, LA0716), *S*. *arcanum* (LA2172), *S*. *huaylense* (LA1983, LA1365)	CARGATCONSENSUS	−631
		CARGCW8GAT	−98, −140
V	*S*. *chmielewskii* (LA2663, LA2695), *S*. *neorickii* (LA2133, CGN24193), *S*. *arcanum* (LA2157)	CARGATCONSENSUS	−631
		CARGCW8GAT	−98, −140, −648
VI	*B*^*og*^ (LA0348*)	CARGATCONSENSUS	−891
		CARGCW8GAT	−139, −328, −493, −510
		CARGNCAT	−492

**NB**: PLACE results of plus (+) strands are considered in each case. The sites # of the elements in PLACE database for reference are S000404 (CARGATCONSENSUS), S000431 (CARGCW8GAT) and S000446 (CARGNCAT)

*Accessions analyzed by EcoTILLING and Sanger sequencing

^**@**^CARGATCONSENSUS: CCWWWWWWGG, CARGCW8GAT: CWWWWWWWWG, CARGNCAT: CCWWWWWWWWGG

**Fig 6:**
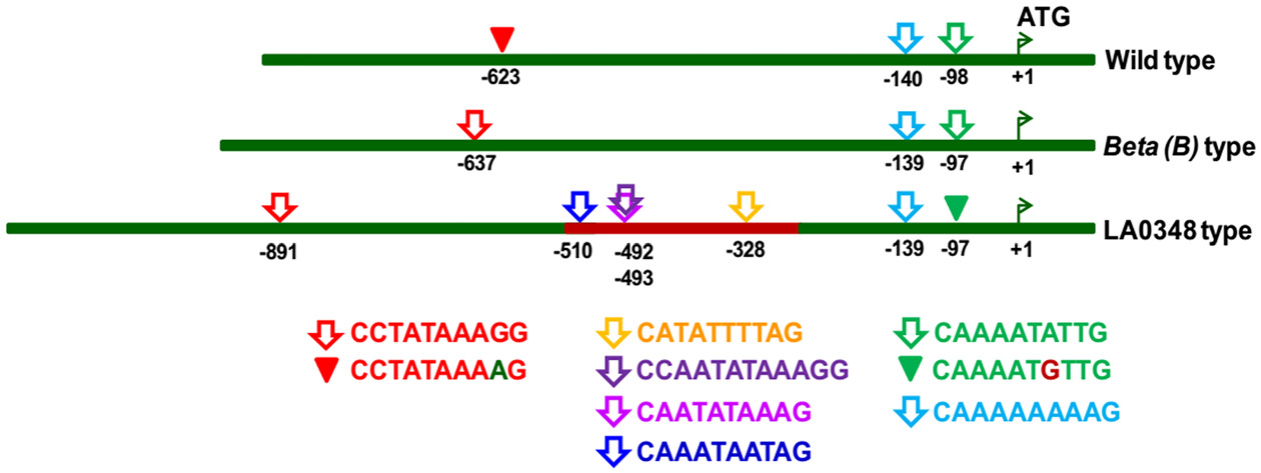
Variability of RIN TF binding sites in *CYC-B* promoters. The nucleotide sequences upstream of *CYC-B* gene in WT, *Beta (B)* type and LA0348 type are diagrammatically represented with green bars. The red coloured portion in LA0348 type represents the probable NUPT fragment. The RIN binding sites are indicated with arrows, and the altered sites are represented by triangles above the bars. The exact position of the first nucleotide in each case is mentioned below the bars. Different RIN binding sequences predicted by PLACE are indicated with different colours. The element CCTATAAAGG is CARGATCONSENSUS. The elements CAAAATATTG, CAAAAAAAAG, CATATTTTAG, CAATATAAAG and CAAATAATAG are CARGCW8GAT type and the element CCAATATAAAGG is CARGNCAT type.

Our transient expression results with three different *CYC-B* promoters and above promoter analysis indicates a probable link between nature and number of ‘CArG’ boxes and promoter efficiency. Both WT and ‘*Beta* type’ promoters have two CARGCW8GAT at −98/−97 (CAAAATATTG) and −139/−140 (CAAAAAAAAG) positions. The CARGATCONSENSUS present in ‘*Beta* type’ (−637) and ‘LA0348 type’ (−891) is CCTATAAAGG, whereas, an altered site is present in WT (CCTATAAAAG) at −623 position. It is likely that the absence of CARGATCONSENSUS makes the WT *CYC-B* promoter less efficient. Moreover, the probable ‘NUPT’ fragment in *CYC-B* promoter in ‘LA0348 type’ has 4 additional sites for RIN binding. They include the 3 CARGCW8GAT boxes- CATATTTTAG (−328), CAATATAAAG (−493) and CAAATAATAG (−510) and a modified CARGNCAT box- CCAATATAAAGG (−492) (“Fig 6”). These additional RIN binding sites may perhaps contribute to the observed higher efficiency of ‘LA0348 type’ promoter than ‘*Beta* type’ promoter.

### CYC-B group of enzymes show higher plastid targeting score than LCYB group

**Fig 7:**
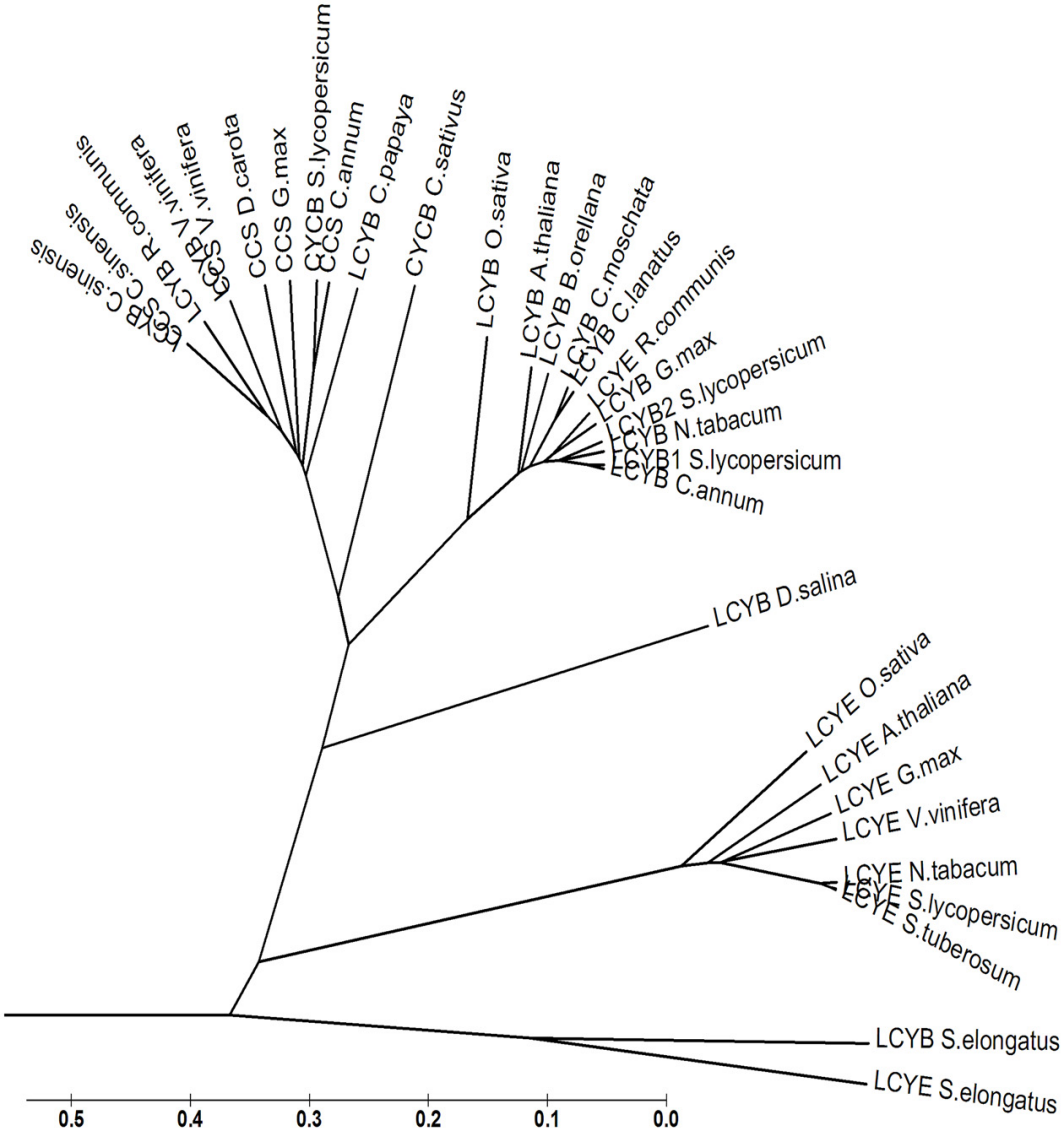
Phylogenic tree showing the similarities between amino acid sequences of CYC-B and its homologues. The analysis involved 32 amino acid sequences. Evolutionary analyses were conducted in MEGA6.

To study the diversity of *CYC-B* gene, the amino acid sequences of various homologues from plant species were obtained from NCBI database through homology search using tomato *CYC-B* gene sequence. The identified homologues included enzymes like LCYB (Lycopene β-cyclase, chloroplastic or common), LCYE (Lycopene ε-cyclase), and CCS (Capsanthin capsorubin synthase). Phylogenetic analysis of CYC-B homologues showed three major groups. First consisted of mostly LCYE (LCYE group), second consisted of mostly chloroplastic LCYB (LCYB group) and third consisted of enzymes like CYC-B, LCYB (common for plastids) and CCS (CYC-B group) (“Fig 7”). Interestingly, LCYB from different plant species showed more homology between themselves than to CYC-B of the same species. It also highlights an early divergence of the CYC-B lineage from the LCYB lineage. Moreover, the gene duplication event seems to have first led to the divergence of LCYE and LCYB group followed by later divergence of chloroplastic LCYB and chromoplastic CYC-B/CCS (“Fig 7”).

Even though, both CYC-B and LCYB perform the same catalytic function; they have highly diverged amino acid sequences (“S10 File”). The alignment of amino acids sequences of CYC-B homologues indicated the maximum divergence in the N-terminal region of protein partly due to high divergence in the transit peptide region (“S11 File”). A comparison of transit peptide of different CYC-B homologues indicated that CYC-B group of enzymes have stronger plastid localization signal (“Table 2”), which could be related to chromoplast specific targeting of CYC-B. These results thus indicate a probable neofunctionalization of the *CYC-B* after its origin from *LCYB*.

**Table 2:** Subcellular localization scores of CYC-B homologues predicted using *‘TargetP 1*.*1’*.

Name*	Amino acid Length	cTP	mTP	SP	other	Locus	RC	Tp length
CYC-B_*Solanum lycopersicum* [AK327886.1]^a^	498	0.895	0.083	0.009	0.048	C	1	42
CYC-B_*Crocus sativus* [GQ202141.1]^a^	468	0.851	0.262	0.01	0.049	C	3	22
LCYB_*Ricinus communis* [EEF30885.1]^a^	495	0.884	0.347	0.004	0.03	C	3	45
LCYB_*Citrus sinensis* [FJ516403.1]^a^	503	0.933	0.03	0.034	0.091	C	1	12
LCYB_*Vitis vinifera* [JQ319643.1]^a^	497	0.937	0.221	0.021	0.032	C	2	42
LCYB_*Carica papaya* [FJ839872.1]^a^	494	0.612	0.109	0.064	0.089	C	3	44
LCYB_*Oryza sativa* [BAD16478.1]^b^	489	0.878	0.212	0.007	0.056	C	2	33
LCYB_*Dunaliella salina* [ACA34344.1]	584	0.861	0.214	0.005	0.064	C	2	68
CCS_*Capsicum annum* [GU122939.1]^a^	498	0.81	0.052	0.03	0.268	C	3	39
CCS_*Vitis vinifera* [XM_002273826.1]^a^	497	0.937	0.221	0.021	0.032	C	2	42
CCS_*Glycine max* [XM_003541917.1]^a^	493	0.764	0.145	0.041	0.114	C	2	41
CCS_*Citrus sinensis* [AF169241.1]^a^	503	0.933	0.03	0.034	0.091	C	1	12
CCS_*Daucus carota* [DQ192191.1]^a^	492	0.089	0.524	0.034	0.475	M	5	58
LCYB1_*Solanum lycopersicum* [AK319553.1]^b^	500	0.26	0.144	0.044	0.553	-	4	-
LCYB2_*Solanum lycopersicum* [AK323472.1]^b^	500	0.205	0.133	0.033	0.609	-	3	-
LCYB_*Capsicum annum* [ADH04277.1]^b^	498	0.188	0.17	0.027	0.542	-	4	-
LCYB_*Nicotiana tabacum* [CAA57386.1]^b^	500	0.188	0.14	0.096	0.638	-	3	-
LCYB_*Bixa orellana* [CAD70565.1]^b^	499	0.259	0.071	0.048	0.534	-	4	-
LCYB_*Arabidopsis thaliana* [NP_187634.1]^b^	501	0.091	0.05	0.075	0.816	-	2	-
LCYB_*Synechococcus elongates* [YP_401079.1]	411	0.058	0.098	0.676	0.473	S	4	17
LCYB_*Cucurbita moschata* [AEN94903.1]^b^	497	0.139	0.308	0.021	0.577	-	4	-
LCYB_*Citrullus lanatus* [ABM90918.1]^b^	504	0.177	0.129	0.023	0.73	-	3	-
LCYB_*Glycine max* [XP_003554131.1]^b^	507	0.385	0.059	0.024	0.713	-	4	-
LCYE_*Solanum lycopersicum* [NP_001234337]^c^	527	0.497	0.305	0.029	0.252	C	5	45
LCYE_*Solanum tuberosum* [DAA33890.1]^c^	527	0.494	0.358	0.029	0.225	C	5	45
LCYE_*Arabidopsis thaliana* [NP_200513.1]^c^	524	0.751	0.442	0.016	0.045	C	4	45
LCYE_*Synechococcus elongates* [ZP_01470358.1]	414	0.039	0.081	0.66	0.578	S	5	22
LCYE_*Ricinus communis* [EEF48090.1]	514	0.119	0.138	0.038	0.761	-	2	-
LCYE_*Glycine max* [XP_003546468.1]^c^	531	0.986	0.341	0.014	0.003	C	2	54
LCYE_*Nicotiana tabacum* [ADZ48238.1]^c^	524	0.312	0.412	0.046	0.223	M	5	9
LCYE_*Vitis vinifera* [AFP28798.1]^c^	530	0.961	0.162	0.023	0.02	C	2	48
LCYE_*Oryza sativa* [BAC05562.1]^c^	540	0.587	0.194	0.067	0.08	C	4	52

*Gene bank/NCBI reference sequence accession numbers are given in bracket.

^a^CYC-B/CCS group,

^b^LCYB group,

^c^LCYE group

**C**: Chloroplast, **M**: Mitochondria, **S**: Secretory pathway, **cTP**: plastid transit peptide, **mTP**: mitochondrial targeting peptide, **SP**: secretory pathway signal peptide, _: Any other location, **RC**: Reliability class [1 indicates the strongest prediction], **TP**: target peptide.

## Discussion

In tomato genome the key genes contributing to carotenoid biosynthesis such as phytoene synthase (*PSY1*, *PSY2*, *PSY3)* and lycopene *β*-cyclase (*LCYB1*, *LCYB2*, *CYC-B*) are triplicated and carry out specific steps related to chloroplast or chromoplast localized carotenogenesis. It is proposed that two consecutive genome triplications in *Solanum* lineage lead to neofunctionalization of genes controlling fruit colour [12]. Normally, during genome evolution duplicated/triplicated genes are lost in a process known as fractionation [31]. However, in tomato clade, the triplicated lycopene *β*-cyclase genes are retained as paralogs and have identical catalytic functions, but underwent regulatory neofunctionalization (R-NF) [32]. Both *PSY1* and *CYC-B* diverged in developmental expression and associated with chromoplast-specific regulation of carotenogenesis during fruit ripening, consistent with the notion that R-NF results from expression divergence in novel temporal and/or spatial environments. The above chromoplast-specific association of *CYC-B* gene is also consistent with the notion that R-NF has a more important role than regulatory subfunctionalization for evolutionary retention and divergence of duplicated genes [33]. In this study, sequence diversity in the promoter and gene sequence of *CYC-B* in tomato and its wild relatives was examined to understand how the sequence divergence contributed to neofunctionalization.

Though all members of tomato clade possess *CYC-B* gene, the ripening specific carotenoid accumulation leading to red/orange fruits is visualized only in tomato and its closest wild relatives, while other members bear green coloured fruits. Consistent with the lack of fruit coloration, the highest SNP frequency in the coding region of *CYC-B* was present in *S*. *huaylense* (LA1364) and *S*. *peruvianum* (LA1954) which are green fruited species. The low SNP diversity in the majority of the colored fruited accessions and the less deleterious nature of the SNPs indicate that the *CYC-B* gene is under stabilizing selection, a notion also statistically supported by a non-significant Tajima’s D and very low Ka/Ks value. The sequence of *CYC-B* gene of tomato is closely similar to *capsanthin/capsorubin synthase* (*CCS*) gene of pepper which also expresses exclusively in chromoplast. However, after duplication of the genome in Solanum ancestor, pepper *CCS* gene underwent both coding and also regulatory neofunctionalization [34]. In pepper, *CCS* gene gained a new function, whereas, in tomato *CYC-B* gene retained its original catalytic activity of lycopene *β*-cyclization. Nevertheless, ethylene-dependent regulation of gene expression during fruit ripening seems to be preserved for both tomato CYC-B and also pepper CCS gene [34].

Tomato genome sequencing revealed that *S*. *lycopersicum* genome has substantial introgression from *S*. *pimpinellifolium* [12, 9]. Such an introgression of *CYC-B* gene from wild relatives is manifested by the presence of a nonsynonymous nucleotide change G868A derived from wild relatives in several tomato accessions (PI 365925, LYC 2962, PI 129097). Moreover, similar to *S*. *pimpinellifolium* these accessions also bear small sized fruits. The almost nil polymorphism observed in *CYC-B* gene in tomato accessions is in conformity with it being under stabilizing selection.

In *CYC-B* gene non-synonymous substitutions (51.7%) are higher than synonymous substitutions, whereas at the whole genome level lower percentage (~45%) is reported for non-synonymous substitutions [9]. Among the analyzed accessions, a green-fruited accession EC20636 showed the maximum number of genetic variations though most of the SNPs had no expected effect on the function of CYC-B protein. Nonetheless, a base change G570A was predicated to cause premature termination of the CYC-B protein with only 189 amino acids. In CYC-B protein, the 293-FLEET-297 motif is highly conserved, and substitution of alanine, lysine, and arginine for glutamate-295 in above motif abolishes the enzyme activity [35]. Therefore, it is unlikely that the truncated CYC-B protein in EC20636 with only 189 amino acids would be enzymatically active. Considering that the green-fruited wild relatives accumulate either nil or very little amount of carotenoids during fruit ripening, a fully functional CYC-B enzyme in these wild relatives does not impart any evolutionary advantage.

Though it is not known whether CYC-B protein is enzymatically active in green-fruited wild relatives, the SIFT scores indicate the presence of deleterious SNPs in few *CYC-B* alleles from wild relatives. In *CYC-B* gene, Lycopersicon group accessions, that bears red/orange fruit has Valine at 23 position while green fruited species Neolycopersicon, Eriopersicon, Arcanum, bears phenylalanine at the same position [9]. It remains to be determined if above substitution and other changes in CYC-B protein affect its activity. The fruit localized carotenoid biosynthesis also requires other regulatory factors such as climacteric regulation of ripening and chloroplast to chromoplast transformation, which is presumably absent in green-fruited wild relatives. Consequently, most of the genetic variations in genes regulating fruit ripening/development are tolerated in green-fruited wild relatives, and higher percentages of SNPs in *CYC-B* gene thus may be related to the absence of carotenogenesis.

A genome-wide examination of genetic polymorphism in -100 tomato accessions including its wild relatives revealed that ~90% of the polymorphism is localized in the intergenic region compared to only ~2% in the exons [9]. Consistent with this, our EcoTILLING analysis of *CYC-B* promoter along with variant sequences obtained from the tomato variant browser revealed that *CYC-B* promoter has more than double nucleotide diversity (π) than the exon. It is plausible that the SNPs and In-Dels in *CYC-B* gene are less tolerated than in the non-coding promoter region, as these may render CYC-B catalytically inactive. Moreover, the changes in the *CYC-B* promoter may be more directed towards R-NF for ripening and/or also a chromoplast-specific expression of *CYC-B* gene. In tomato, similar to *CYC-B*, two paralogous genes- *Bl* and *C* regulate branching and leaflet initiation respectively. It is proposed that functional differentiation of these two genes is due to different promoter activity presumably by the divergence in promoter sequences [36]. The changes during tomato clade evolution that lead to neofunctionalization of *CYC-B* promoter restricting its expression during fruit ripening are not known. In tomato post mature green stage several transcriptional factors such as TAGL, NOR, FUL and RIN regulates ripening. While changes in the *CYC-B* promoter sequence may have contributed to its fruit-specific expression, the species specific differential expression of promoter may be related to the presence/absence of ripening regulators.

Considering that an introgression in the promoter region of *B* allele from a wild relative leads to higher expression of *CYC-B* gene [4], it is plausible that the NUPT fragment in the *CYC-B* promoter region of the accessions (LA0348, LA0500-accessions with *B*^*og*^ mutation) and LA0458 (*S*. *chilense*) may similarly affect the expression of *CYC-B*. In conformity with above view, the transient expression of ‘*Beta* type’ and ‘LA0348 type’ promoters showed that these two promoters are more efficient than WT with ‘LA0348 type’ promoter being most efficient. Apparently the insertion of NUPT fragment may have increased the efficiency of ‘LA0348 type’ *CYC-B* promoter. In plants, shuffling of genome fragments between organelles and nucleus have been reported in several species [37–39]. In tomato genome, nearly 1513 nuclear plastid DNAs (NUPT) are present contributing to 0.084% of total genomic DNA [40]. It is reported that functionally important NUPTs are rare, and most NUPT are deleterious and get eliminated from genome [40]. The retention of the NUPT fragment in the *CYC-B* promoter of the *B*^*og*^ mutants appears to be an isolated event. It must be however considered that source of ‘LA0348 type’ promoter was a *S*. *chilense* accession that was used for crossing with *S*. *lycopersicum* [5, 6], wherein ripening induced carotenoid accumulation is not observed. However, the lack of carotenoid accumulation is not related to the efficiency of ‘LA0348 type’ promoter. *B*^*og*^ mutation was identified as a spontaneous mutation in one of the F2 lines (out of 15 F2 lines) during the crossing, and thus was a novel loss of function *CYC-B* mutant [5].

In tomato, evidences support that transcription factor RIN (Ripening INhibitor) act as a master regulator to induce the ripening [41, 30]. It is believed that RIN acts by binding at “CArG” box post-mature-green stage of fruit ripening in tomato. The promoter of the *CYC-B* genes in the majority of tomato accessions and its closest wild relative S. *pimpinellifolium* carry only two CArG boxes present at -97/98 and -139/140 position. In few tomato accessions and/or wild relatives, either additional CArG like boxes (CARGATCONSENSUS and/or CARGNCAT) are present, and/or location of the CArG like boxes are different. Since the majority of red fruited tomato accessions do not have CARGATCONSENSUS box, it may be concluded that the above element was lost during evolution.

The R-NF of *CYC-B* gene entails that during the evolution of tomato clade, its promoter activity was restricted to fruit ripening phase. Enhanced expression of *CYC-B* gene during fruit ripening leads to higher accumulation of *β*-carotene resulting in orange colored fruits in tomato ‘*Beta*’ mutant [4]. Considering that the promoter region of ‘*Beta*’ mutant has an extra CARGATCONSENSUS box providing additional RIN binding site, its overexpression appears to be related to above additional element. Consistent with this notion, ‘*Beta* type’ promoter showed higher transient expression than WT. The higher expression of ‘LA0348 type’ promoter thus may be related to the presence of similar extra elements in the promoter.

It seems that the appearance of chromoplast differentiation was associated with attenuation of *CYC-B* expression, as both tomato and its close wild relative *S*. *pimpinellifolium* have only two CArG boxes. It is likely that the presence of an SNP in the RIN binding site in tomato may be responsible for the reduction in the *CYC-B* expression. The loss of CARGATCONSENSUS box sequence from *CYC-B* promoter may be thus required for attenuation of gene expression. This possibility needs to be verified through additional experiments. At the same time, other regulatory elements such as light and hormonal signalling elements, present in *CYC-B* promoter may influence its expression during ripening [42]. It is reported that climacteric rise of ethylene during tomato fruit ripening inhibits *CYC-B* expression leading to preferential accumulation of lycopene in fruits [43]. The reduction in *CYC-B* expression may also be associated with preferential accumulation of lycopene in tomato fruits for red colour to assist foraging by birds [44].

The genes coding for LCYB, CYC-B, CCS and LCYE appear to be evolved from an ancestral lycopene cyclase gene through gene duplications and acquisition of specialized functions [45]. For the origin of *CYC-B* and *CCS*, the probability of a retrotransposon-mediated event from an ancestral *LCYB* gene is also predicted as both lack introns unlike *LCYB* [4]. Subsequently, in pepper, *CCS* gene underwent coding neofunctionalization to acquire a specialized function with different substrate specificities [46, 34]. The strong plastid targeting signal of CYC-B compared to that of LCYB in tomato may have also contributed to the functional specialization of CYC-B in chromoplastic tissues. The chromoplast specific localization of CYC-B is also consistent with the notion that protein neolocalization is a key event in neofunctionalization of duplicated genes [47]. This also indicates a second NF of CYC-B at post-translational levels for efficient targeting and/or function in chromoplast.

The observed pattern of transition and transversion rate in the *CYC-B* promoter sequences might be related to the methylation pattern. In coloured fruited species fruit ripening is associated with upregulation of the carotenoid biosynthesis pathway genes. In general, hypermethylation suppresses fruit ripening process since the binding of transcription factors requires a demethylated state of the promoter sequences to activate transcription of the ripening associated genes [48]. It is likely that in green-fruited species the methylation of the promoter may have affected transcriptional activity of ripening-related genes including *CYC-B*. The methylation also increases the possibility of spontaneous demethylation process leading to a cytosine (C) to thymine (T) conversion. This might be the reason for the higher rate of transition in the promoters of green fruited species compared to coloured fruited species; however other possibilities also exists for the higher transition.

In summary, our results indicate that both neolocalization to chromoplasts and R-NF of *CYC-B* may have played an important role in the regulation of carotenoid biosynthesis during tomato fruit ripening. In tomato, *CYC-B* gene shows little polymorphism perhaps due to strong selection by humans during domestication. The observed polymorphism in *CYC-B* gene and its promoter are mainly from introgression from wild relatives of tomato. While our results are indicative of a role for R-NF in regulating carotenoid biosynthesis in tomato fruits, a more intensive study is needed involving other genes and regulatory partners, to understand the role of neofunctionalization during tomato fruit evolution.

## Conclusions

In tomato clade, little information is available about factors contributing to neofunctionalization of genes controlling fruit colour. The appearance of chromoplast differentiation was associated with attenuation of *CYC-B* expression. The beneficial effect of introgression of NUPT fragment in CYC-B promoter represents a rare case of the gain of function by NUPT insertion. The strong plastid targeting signal in CYC-B likely contributed to chromoplast-specific localization of CYC-B. Our study indicates that neofunctionalization of *CYC-B* and neolocalization to chromoplasts played an important role in chromoplast- specific carotenogenesis in tomato fruits.

## Supporting Information

S1 File List of tomato accessions used for EcoTILLING. (XLSX)

S2 File Details of the primers designed for EcoTILLING through nested PCR strategy (Universal M13 sequences are indicated in bold letters). (DOCX)

S3 File List of accessions from the 'Tomato structural variation browser'. (XLSX)

S4 File Distribution of SNPs and In-dels in *CYC-B* gene from various accessions. A total of 84 variant sites are identified in *CYCB* gene through EcoTILLING (22) and from Variant Browser (33) out of which 28 are common in both sets. Out of 484 total accessions examined, 54 accessions are identified with variant sites which include 19 from EcoTILLING and 37 from Variant Browser with two common accessions (*S*. *neorickii*; LA21330 and *S*. *pennellii*; LA0716). The 33 sites from Variant Browser belonged to 21 out of 37 accessions. Remaining 16 out of 37 accessions shared few of the polymorphism with EcoTILLING population. Presence of variants are indicated with colored boxes corresponding to the accession and the change in nucleotide. Color of the boxes indicate the group to which the accession belongs. Red: Lycopersicon, Dark green: Eriopersicon, Blue: Arcanum, Purple: Neolycopersicon, Light green: Unidentified. *symbol indicates accessions analyzed by EcoTILLING and Sanger sequencing.
(PDF)

S5 File List of SNPs in *CYC-B* gene and their probable effects predicted based on SIFT score. (DOCX)

S6 File List of haplotypes identified for *CYC-B* gene using DnaSP software. (DOCX)

S7 File Distribution of SNPs in *CYC-B* gene from various haplotypes. The exon (1–1497 bp) and 3´UTR (1498–1667 bp) of the gene is diagrammatically represented with bar and line respectively. Black, purple and red triangles indicate the positions of nonsynonymous, synonymous and nonsense nucleotide substitutions respectively. Red squares indicate the positions of In-dels. Haplotype 1 is reference haplotype.
(PDF)

S8 File List of SNPs with the respective changes in restriction enzyme sites (predicted by PARSESNP). (DOCX)

S9 File List of haplotypes identified for *CYC-B* promoter using DnaSP software. (DOCX)

S10 File Amino acid sequence identities (%) between CYC-B homologues (NCBI blastp suite-2sequences). (DOCX)

S11 File Alignment of amino acid sequences of CYC-B and its homologues. Multalin software (http://multalin.toulouse.inra.fr/multalin/multalin.html) (Corpet, 1988) was used for making the alignment. Red color indicates high consensus (>90%) and blue color indicates low consensus (<50%) amino acid residues. The N-terminal region of all the enzymes are highly diverged.
(PDF)
