# Supplementary material for: Neofunctionalization of Chromoplast Specific Lycopene Beta Cyclase Gene (CYC-B) in Tomato Clade
Source: PLoS One. 2016 Apr 12;11(4):e0153333. doi: 10.1371/journal.pone.0153333 (PMC4829152; doi:10.1371/journal.pone.0153333)
Supplement: S2 File — (DOCX) [file pone.0153333.s002.docx]

|  | **Primer Name** | | | **Sequence 5´-3´** | | **Length**  **(bp)** | |
| --- | --- | --- | --- | --- | --- | --- | --- |
| I step | | NF | CTGATTGTGCTCTTCCTTTACTTG | | 24 | |  |
|  |  | NR | GAAAAGACACAAGCTGAGTAAACC | | 24 | |  |
| Promoter | | PNF | **TGTAAAACGACGGCCAGT**TCCACAGTGAGCATTTCGATCTAC | | 42 | |  |
|  |  | PNR | **AGGAAACAGCTATGACCAT**TGGGTGCTAAATCAAGAAAGCTAC | | 43 | |  |
| Exon | | ENF | **TGTAAAACGACGGCCAGT**CTCTTCTCAAGCCTTTTCCATCTC | | 42 | |  |
|  |  | ENR | **AGGAAACAGCTATGACCAT**CTAACACATCTTCTATCCAAAGGC | | 43 | |  |
| Universal | | M13F | **IRD_700_-TGTAAAACGACGGCCAGT** | | 18 | |  |
|  |  | M13R | **IRD_800_-AGGAAACAGCTATGACCAT** | | 19 | |  |
