# Supplementary material for: Neofunctionalization of Chromoplast Specific Lycopene Beta Cyclase Gene (CYC-B) in Tomato Clade
Source: PLoS One. 2016 Apr 12;11(4):e0153333. doi: 10.1371/journal.pone.0153333 (PMC4829152; doi:10.1371/journal.pone.0153333)
Supplement: S4 File — A total of 84 variant sites are identified in CYCB gene through EcoTILLING (22) and from Variant Browser (33) out of which 28 are common in both sets. Out of 484 total accessions examined, 54 accessions are identified with variant sites which include 19 from EcoTILLING and 37 from Variant Browser with two common accessions (S. neorickii; LA21330 and S. pennellii; LA0716). The 33 sites from Variant Browser belonged to 21 out of 37 accessions. Remaining 16 out of 37 accessions shared few of the polymorphism with EcoTILLING population. Presence of variants are indicated with colored boxes corresponding to the accession and the change in nucleotide. Color of the boxes indicate the group to which the accession belongs. Red: Lycopersicon, Dark green: Eriopersicon, Blue: Arcanum, Purple: Neolycopersicon, Light green: Unidentified. *symbol indicates accessions analyzed by EcoTILLING and Sanger sequencing. (PDF) [file pone.0153333.s004.pdf]

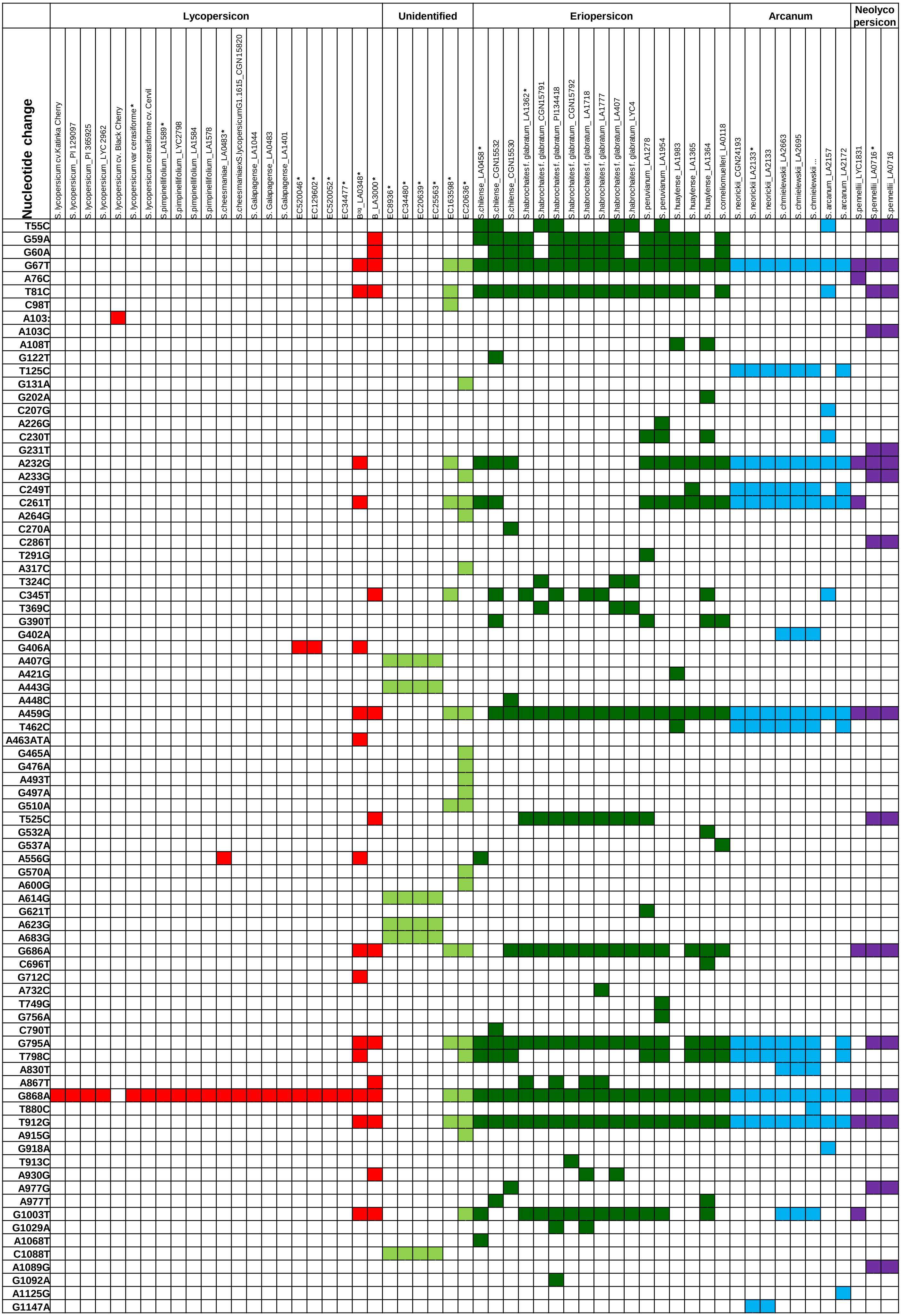
Nucleotide change
Lycopersicon
Unidentified
Eriopersicon
Arcanum
Neolyco persicon
S. lycopersicum cv.Katinka Cherry
S. lycopersicum_PI 129097
S. lycopersicum_PI 365925
S. lycopersicum_LYC 2962
S. lycopersicum cv. Black Cherry
S. lycopersicum var cerasiforme*
S. lycopersicum cerasiforme cv. Cervil
S.pimpinellifolium_LA1589*
S.pimpinellifolium_LYC2798
S.pimpinellifolium_LA1584
S.pimpinellifolium_LA1578
S.cheesmaniae_LA0483*
S.cheesmaniaexS.lycopersicumG1.1615_CGN15820
S. Galapagense_LA1044
S. Galapagense_LA0483
S. Galapagense_LA1401
EC520046*
EC129602*
EC520052*
EC34477*
B_LA0348*
B_LA3000*
EC8936*
EC34480*
EC20639*
EC25563*
EC163598*
EC20636*
S.chilense_LA0458*
S.chilense_CGN15532
S.chilense_CGN15530
S.habrochaites f. glabratum_LA1362*
S.habrochaites f. glabratum_CGN15791
S.habrochaites f. glabratum_PI134418
S.habrochaites f. glabratum_ CGN15792
S.habrochaites f. glabratum_ LA1718
S.habrochaites f. glabratum_LA1777
S.habrochaites f. glabratum_LA407
S.habrochaites f. glabratum_LYC4
S. peruvianum_LA1278
S. peruvianum_LA1954
S. huaylense_LA1983
S. huaylense_LA1365
S. huaylense_LA1364
S. corneliomuelleri_LA0118
S. neorickii_CGN24193
S. neorickii LA2133*
S. neorickii_LA2133
S. chmielewskii_LA2663
S. chmielewskii_LA2695
S. chmielewskii...
S. arcanum_LA2157
S. arcanum_LA2172
S.pennellii_LYC1831
S.pennellii_LA0716*
S.pennellii_LA0716
T55C
G59A
G60A
G67T
A76C
T81C
C98T
A103:
A103C
A108T
G122T
T125C
G131A
G202A
C207G
A226G
C230T
G231T
A232G
A233G
C249T
C261T
A264G
C270A
C286T
T291G
A317C
T324C
C345T
T369C
G390T
G402A
G406A
A407G
A421G
A443G
A448C
A459G
T462C
A463ATA
G465A
G476A
A493T
G497A
G510A
T525C
G532A
G537A
A556G
G570A
A600G
A614G
G621T
A623G
A683G
G686A
C696T
G712C
A732C
T749G
G756A
C790T
G795A
T798C
A830T
A867T
G868A
T880C
T912G
A915G
G918A
T913C
A930G
A977G
A977T
G1003T
G1029A
A1068T
C1088T
A1089G
G1092A
A1125G
G1147A
