# Supplementary material for: Neofunctionalization of Chromoplast Specific Lycopene Beta Cyclase Gene (CYC-B) in Tomato Clade
Source: PLoS One. 2016 Apr 12;11(4):e0153333. doi: 10.1371/journal.pone.0153333 (PMC4829152; doi:10.1371/journal.pone.0153333)
Supplement: S5 File — (DOCX) [file pone.0153333.s005.docx]

| **S No** | **Nucleotide Change** | **Effect** | **SIFT Score^$^** | **Description** |
| --- | --- | --- | --- | --- |
| **1** | T55C | Y19H | 0.54 | Tolerated |
| **2** | G59A | R20K | 0.95 | Tolerated |
| **3** | G60A | R20= |  |  |
| **4** | G67T | V23F | 0.72 | Tolerated |
| **5** | A76C | N26H | 0.14 | Tolerated |
| **6** | T81C | P27= |  |  |
| **7** | C98T | T33I | 0.31 | Tolerated |
| **8** | A103: | Frameshift |  | **Truncation** |
| **9** | A103C | K35Q | 0.62 | Tolerated |
| **10** | A108T | K36N | 0.51 | Tolerated |
| **11** | G122T | C41F | 0.76 | Tolerated |
| **12** | T125C | L42P | 0.38 | Tolerated |
| **13** | G131A | R44K | 1 | Tolerated |
| **14** | G202A | V68I | 0.56 | Tolerated |
| **15** | C207G | N69K | 0.25 | Tolerated |
| **16** | A226G | N76D | 0.69 | Tolerated |
| **17** | C230T | S77L | 0.52 | Tolerated |
| **18** | G231T | S77= |  |  |
| **19** | A232G | N78D | 0 | Tolerated |
| **20** | A233G | N78S | 0.73 | Tolerated |
| **21** | C249T | D83= |  |  |
| **22** | C261T | I87= |  |  |
| **23** | A264G | G88= |  |  |
| **24** | C270A | G90= |  |  |
| **25** | C286T | L96= |  |  |
| **26** | T291G | A97= |  |  |
| **27** | A317C | K106T | 0.27 | Tolerated |
| **28** | T324C | C108= |  |  |
| **29** | C345T | L115= |  |  |
| **30** | T369C | G123= |  |  |
| **31** | G390T | E130D | 0.33 | Tolerated |
| **32** | G402A | L134= |  |  |
| **33** | G406A | D136N | 0 | Tolerated |
| **34** | A407G | D136G | 0.22 | Tolerated |
| **35** | A421G | K141E | 0.37 | Tolerated |
| **36** | A443G | H148R | **0.03** | **Affect protein function** |
| **37** | A448C | N150H | 0.07 | Tolerated |
| **38** | A459G | K153= |  |  |
| **39** | T462C | T154= |  |  |
| **40** | A463ATA | Frameshift |  | **Truncation** |
| **41** | G465A | K155= |  |  |
| **42** | G476A | R159K | 0.11 | Tolerated |
| **43** | A493T | S165C | **0.02** | **Affect protein function** |
| **44** | G497A | R166K | **0** | **Affect protein function** |
| **45** | G510A | K170= |  |  |
| **46** | T525C | N175= |  |  |
| **47** | G532A | V178I | 0.71 | Tolerated |
| **48** | G537A | E179= |  |  |
| **49** | A556G | K186E | 0.83 | Tolerated |
| **50** | G570A | W190* |  | **Truncation** |
| **51** | A600G | S200= |  |  |
| **52** | A614G | D205G | 0.44 | Tolerated |
| **53** | G621T | K207N | 0.45 | Tolerated |
| **54** | A623G | K208R | 0.52 | Tolerated |
| **55** | A683G | D228G | **0.04** | **Affect protein function** |
| **56** | G686A | R229K | 1 | Tolerated |
| **57** | C696T | N232= |  |  |
| **58** | G712C | A238P | **0** | **Affect protein function** |
| **59** | A732C | E244D | 0.05 | Tolerated |
| **60** | T749G | F250C | **0.01** | **Affect protein function** |
| **61** | G756A | L252= |  |  |
| **62** | C790T | H264Y | 0 | Tolerated |
| **63** | G795A | L265= |  |  |
| **64** | T798C | G266= |  |  |
| **65** | A830T | K277I | **0.04** | **Affect protein function** |
| **66** | A867T | R289S | 0.91 | Tolerated |
| **67** | G868A | D290N | 1 | Tolerated |
| **68** | T880C | L294= |  |  |
| **69** | T912G | V304= |  |  |
| **70** | T913C | L305= |  |  |
| **71** | A915G | L305= |  |  |
| **72** | G918A | S306= |  |  |
| **73** | A930G | V310= |  |  |
| **74** | A977G | K326R | 0 | Tolerated |
| **75** | A977T | K326I | **0.03** | **Affect protein function** |
| **76** | G1003T | V335L | 0.77 | Tolerated |
| **77** | G1029A | P343= |  |  |
| **78** | A1068T | S356= |  |  |
| **79** | C1088T | T363I | **0.01** | **Affect protein function** |
| **80** | A1089G | T363= |  |  |
| **81** | G1092A | G364= |  |  |
| **82** | A1125G | P375= |  |  |
| **83** | G1147A | E383K | 0.58 | Tolerated |

**^$^**SIFT: Sorting Intolerant From Tolerant (Score ranges from 0 to 1, where 0 is damaging and 1 is neutral, <0.05: deleterious).
