# Supplementary material for: Neofunctionalization of Chromoplast Specific Lycopene Beta Cyclase Gene (CYC-B) in Tomato Clade
Source: PLoS One. 2016 Apr 12;11(4):e0153333. doi: 10.1371/journal.pone.0153333 (PMC4829152; doi:10.1371/journal.pone.0153333)
Supplement: S6 File — (DOCX) [file pone.0153333.s006.docx]

| **Haplotype** | **No. of accessions** | **Accessions** | **Specific SNPs^$^** | **Group^@^** |
| --- | --- | --- | --- | --- |
| Hap_1 | 532 | *S. lycopersicum* cv. Arka Vikas*, *S. lycopersicum* cv. Heinz_1706 and all other accessions in the study | - | Lycopersicon |
| Hap_2 | 1 | *S. peruvianum*_LA1278 | T291G, G621T | Eriopersicon |
| Hap_3 | 1 | *S. peruvianum*_LA1954 | A226G |  |
|  |  |  | T749G**^#^** |  |
|  |  |  | G756A |  |
| Hap_4 | 1 | *S. corneliomuelleri*_LA0118 | G537A |  |
| Hap_5 | 1 | *S. huaylense*_LA1365 | - |  |
| Hap_6 | 1 | *S. chilense*_CGN15532 | G122T, C790T**^#^** |  |
| Hap_7 | 1 | *S. chilense*_CGN15530 | C270A |  |
|  |  |  | A448C |  |
| Hap_8 | 1 | *S. huaylense*_LA1364 | G202A |  |
|  |  |  | G532A |  |
|  |  |  | C696T |  |
| Hap_9 | 1 | EC163598* | C98T | - |
| Hap_10 | 2 | *S. neorickii*_LA2133*, | G1147A | Arcanum |
|  |  | *S. neorickii*_LA2133 |  |  |
| Hap_11 | 1 | *S. neorickii*_CGN24193 | - |  |
| Hap_12 | 1 | *S. arcanum*_LA2172 | A1125G |  |
| Hap_13 | 2 | *S. chmielewskii*_LA2663, *S. chmielewskii*_LA2695 | - |  |
| Hap_14 | 1 | *S. chmielewskii* | T880C |  |
| Hap_15 | 1 | *S. habrochaites f.glabratum*_LA1362* | - | Eriopersicon |
| Hap_16 | 1 | *S. habrochaites f.glabratum*_LA0407 | - |  |
| Hap_17 | 1 | *B*_LA3000* | - | Lycopersicon |
| Hap_18 | 1 | *S. habrochaites f.glabratum*_PI134418 | G1092A | Eriopersicon |
| Hap_19 | 1 | *S. habrochaites f.glabratum*_CGN15792 | T913C |  |
| Hap_20 | 1 | *S. habrochaites f.glabratum*_LA1718 | - |  |
| Hap_21 | 2 | *S. habrochaites f.glabratum*_CGN15791, *S. habrochaites f*.*glabratum*_LYC4 | - |  |
| Hap_22 | 1 | *S. habrochaites f.glabratum*_LA1777 | A732C |  |
| Hap_23 | 1 | *S. pennellii*_LA0716*, *S. pennellii*_LA0716 | A103C, G231T | Neolycopersicon |
|  |  |  |  |  |
| Hap_24 | 4 | EC20639*, EC8936*, EC34480*, EC25563* | A407G | - |
|  |  |  | A443G**^#^** |  |
|  |  |  | A614G |  |
|  |  |  | A623G |  |
|  |  |  | A683G**^#^** |  |
|  |  |  | C1088T**^#^** |  |
| Hap_25 | 1 | cv. Black Cherry | A103:**^##^** | Lycopersicon |
| Hap_26 | 17 | *S. lycopersicum*_PI365925, *S. lycopersicum*_LYC2962, *S. lycopersicum*_PI129097, cv. Katinka Cherry, *S. lycopersicum cerasiforme* cv. Cervil, *S. galapagense*_LA1401, *S. galapagense*_LA0483, *S. galapagense*_LA1044, *S. Cheesmaniae x S. lycopersicum G1.1615(CGN15820), S. pimpinellifolium*_LYC2798, *S. pimpinellifolium*_LA1584, *S. pimpinellifolium*_LA1578, *S. cheesmaniae*_LA0483*, *S. pimpinellifolium*_LA1589*, EC520052, EC34477, *cerasiforme* | - |  |
| Hap_27 | 2 | EC520046*, EC129602* | - |  |
| Hap_28 | 1 | *S. arcanum_*LA2157 | C207G | Arcanum |
|  |  |  | G918A |  |
| Hap_29 | 1 | *S. huaylense*_LA1983 | A421G | Eriopersicon |
| Hap_30 | 1 | *S. pennellii*_LYC1831 | A76C | Neolycopersicon |
| Hap_31 | 1 | *S. chilense*_LA0458* | A1068T | Eriopersicon |
| Hap_32 | 1 | EC20636* | G131A, A264G, A317C, G465A, G476A, A493T**^#^** G497A**^#^** G570A**^###^** A600G, A915G | - |
| Hap_33 | 1 | *B^og^*_LA0348* | A463ATA**^##^** | Lycopersicon |
|  |  |  | G712C**^#^** |  |

^*^Analyzed by EcoTILLING and Sequencing by Sanger method

**^$^**SNPs which are exclusively present in each haplotype

**^#^**Deleterious: predicted to affect protein function by SIFT

**^##^**Highly deleterious: causes truncation of the enzyme (previously reported)

**^###^**Highly deleterious: causes truncation of the enzyme (novel)
