# Supplementary figures and images for: Neofunctionalization of Chromoplast Specific Lycopene Beta Cyclase Gene (CYC-B) in Tomato Clade
Source: PLoS One. 2016 Apr 12;11(4):e0153333. doi: 10.1371/journal.pone.0153333 (PMC4829152; doi:10.1371/journal.pone.0153333)

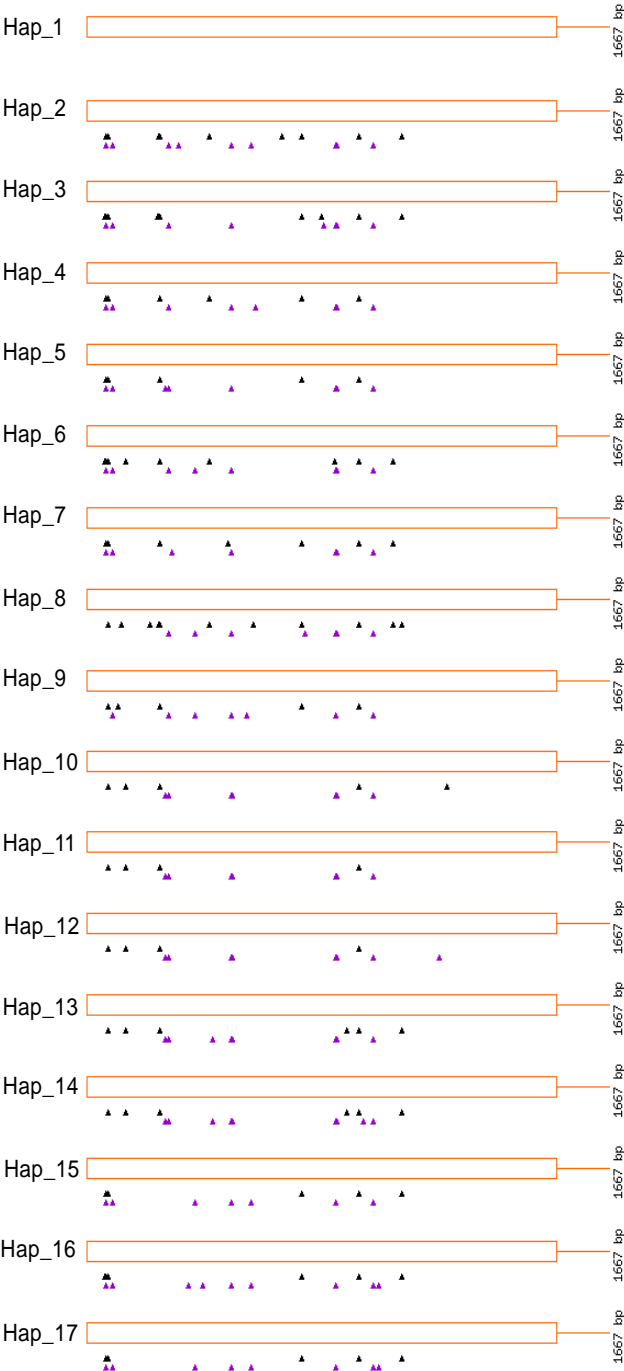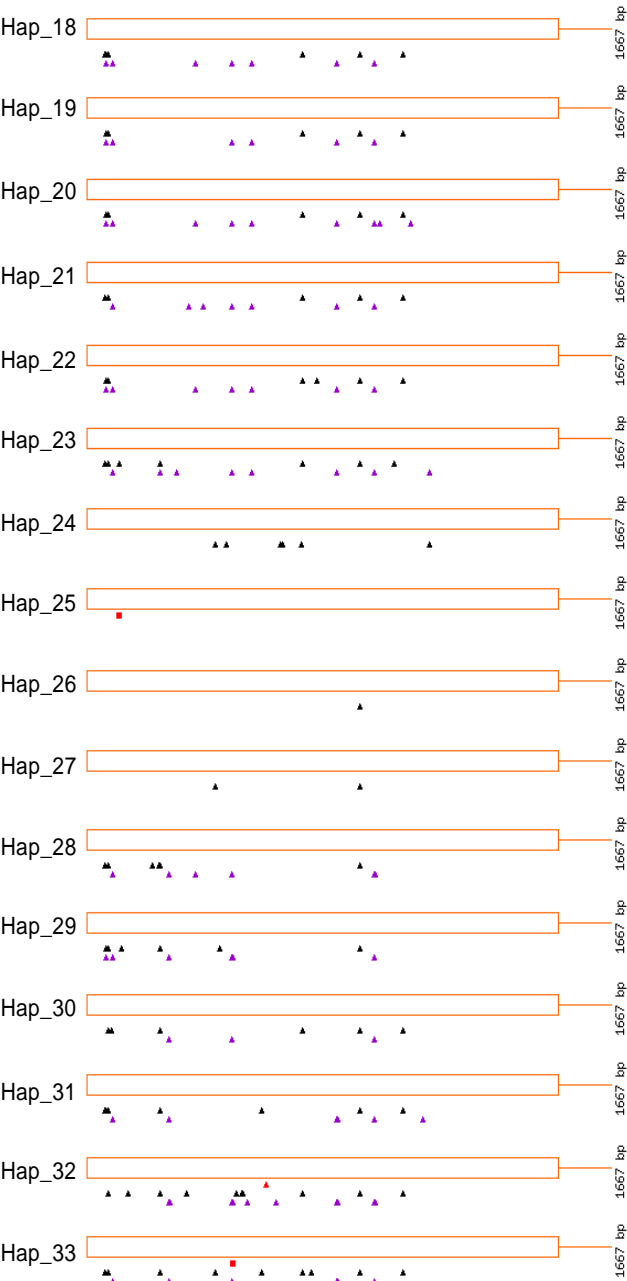

Supplement: S7 File — The exon (1–1497 bp) and 3´UTR (1498–1667 bp) of the gene is diagrammatically represented with bar and line respectively. Black, purple and red triangles indicate the positions of nonsynonymous, synonymous and nonsense nucleotide substitutions respectively. Red squares indicate the positions of In-dels. Haplotype 1 is reference haplotype. (PDF) [file pone.0153333.s007.pdf]
