# Supplementary material for: Neofunctionalization of Chromoplast Specific Lycopene Beta Cyclase Gene (CYC-B) in Tomato Clade
Source: PLoS One. 2016 Apr 12;11(4):e0153333. doi: 10.1371/journal.pone.0153333 (PMC4829152; doi:10.1371/journal.pone.0153333)
Supplement: S8 File — (DOCX) [file pone.0153333.s008.docx]

| **Nucleotide Change** | **Restriction Enzyme Differences from REBASE** | |
| --- | --- | --- |
|  | **Gained in Variant** | **Lost from Reference** |
| T55C |  | [SfeI](http://rebase.neb.com/rebase/enz/SfeI.html) |
| G59A |  | [SfeI](http://rebase.neb.com/rebase/enz/SfeI.html) |
| G60A | BglII, DpnI, Hin4I, MboI, XhoII |  |
| G67T |  |  |
| A76C | [FokI](http://rebase.neb.com/rebase/enz/FokI.html) |  |
| T81C | [MnlI](http://rebase.neb.com/rebase/enz/MnlI.html) |  |
| C98T | BccI, HphI |  |
| A103: |  |  |
| A103C | [TaqII](http://rebase.neb.com/rebase/enz/TaqII.html) |  |
| A108T |  |  |
| G122T | ApoI, TspEI | [BbvII](http://rebase.neb.com/rebase/enz/BbvII.html) |
| T125C | [MnlI](http://rebase.neb.com/rebase/enz/MnlI.html) | BbvII, MboII |
| G131A | [MseI](http://rebase.neb.com/rebase/enz/MseI.html) | [DdeI](http://rebase.neb.com/rebase/enz/DdeI.html) |
| G202A |  | AccI, HindII, HpaI, MjaIV |
| C207G | BglII, DpnI, MboI, XhoII | AccI, HindII, HpaI, MjaIV |
| A226G | BsaBI, HinfI, Hpy178III, TfiI | [TspEI](http://rebase.neb.com/rebase/enz/TspEI.html) |
| C230T |  | AsuII, TaqI |
| G231T |  | AsuII, HinfI, TaqI, TfiI |
| A232G | BinI, DpnI, Hpy188I, MboI | AsuII, HinfI, TaqI, TfiI |
| A233G | BplI, PleI | AsuII, TfiI |
| C249T |  | BtrI, Hpy99I, MaeII |
| C261T |  | BsaBI, Hpy188I |
| A264G |  | AluI, Hpy188I |
| C270A | AvaII, BspMI | Cac8I, CviJI, HaeIII |
| C286T |  | Cac8I, CviJI, MaeI, NheI |
| T291G | [AciI](http://rebase.neb.com/rebase/enz/AciI.html) | AluI, CviJI |
| A317C | [Tsp4CI](http://rebase.neb.com/rebase/enz/Tsp4CI.html) | [MseI](http://rebase.neb.com/rebase/enz/MseI.html) |
| T324C |  |  |
| C345T |  |  |
| T369C |  |  |
| G390T |  | [ApoI](http://rebase.neb.com/rebase/enz/ApoI.html) |
| G402A | [MaeI](http://rebase.neb.com/rebase/enz/MaeI.html) | [BsrI](http://rebase.neb.com/rebase/enz/BsrI.html) |
| G406A | [TspEI](http://rebase.neb.com/rebase/enz/TspEI.html) | [MboII](http://rebase.neb.com/rebase/enz/MboII.html) |
| A407G |  | [MboII](http://rebase.neb.com/rebase/enz/MboII.html) |
| A421G | BspHI, Hpy178III, NlaIII, TspDTI |  |
| A443G |  | CviRI, MslI |
| A448C | [NlaIII](http://rebase.neb.com/rebase/enz/NlaIII.html) |  |
| A459G |  |  |
| T462C | [BstXI](http://rebase.neb.com/rebase/enz/BstXI.html) | [DdeI](http://rebase.neb.com/rebase/enz/DdeI.html) |
| A463ATA |  | [DdeI](http://rebase.neb.com/rebase/enz/DdeI.html) |
| G465A | [SspI](http://rebase.neb.com/rebase/enz/SspI.html) | [DdeI](http://rebase.neb.com/rebase/enz/DdeI.html) |
| G476A |  | BbvII, MboII |
| A493T |  |  |
| G497A |  |  |
| G510A | [TspEI](http://rebase.neb.com/rebase/enz/TspEI.html) | Eco57I, Eco57MI |
| T525C | [Tsp4CI](http://rebase.neb.com/rebase/enz/Tsp4CI.html) |  |
| G532A |  |  |
| G537A |  |  |
| A556G | [TspDTI](http://rebase.neb.com/rebase/enz/TspDTI.html) | [PsiI](http://rebase.neb.com/rebase/enz/PsiI.html) |
| G570A |  |  |
| A600G | [TaqI](http://rebase.neb.com/rebase/enz/TaqI.html) | MfeI, TspEI |
| A614G |  |  |
| G621T |  | [MboII](http://rebase.neb.com/rebase/enz/MboII.html) |
| A623G | [MnlI](http://rebase.neb.com/rebase/enz/MnlI.html) | [MboII](http://rebase.neb.com/rebase/enz/MboII.html) |
| A683G | [Cac8I](http://rebase.neb.com/rebase/enz/Cac8I.html) |  |
| G686A |  | [HaeIII](http://rebase.neb.com/rebase/enz/HaeIII.html) |
| C696T |  | DsaI, NcoI, SecI, StyI |
| G712C | ApoI, MnlI |  |
| A732C | AccI, MaeII, MjaIV |  |
| T749G | MslI, NlaIII |  |
| G756A |  |  |
| C790T |  | [BplI](http://rebase.neb.com/rebase/enz/BplI.html) |
| G795A |  |  |
| T798C | [BsrDI](http://rebase.neb.com/rebase/enz/BsrDI.html) |  |
| A830T | [SfeI](http://rebase.neb.com/rebase/enz/SfeI.html) |  |
| A867T |  |  |
| G868A | ApoI, TspEI |  |
| T880C | EcoRII, PfoI, ScrFI |  |
| T912G |  |  |
| T913C |  |  |
| A915G |  |  |
| G918A |  |  |
| A930G |  |  |
| A977G |  |  |
| A977T |  |  |
| G1003T |  |  |
| G1029A | [TspGWI](http://rebase.neb.com/rebase/enz/TspGWI.html) | AciI, FnuDII, NspBII, SacII |
| A1068T |  |  |
| C1088T |  |  |
| A1089G |  |  |
| G1092A | BciVI, TstI | [RsaI](http://rebase.neb.com/rebase/enz/RsaI.html) |
| A1125G | AgeI, BetI, Cfr10I, HpaII | BsrI, ScaI, TatI |
| G1147A |  | Hpy99I, MnlI, TaqI |
