# Supplementary material for: Neofunctionalization of Chromoplast Specific Lycopene Beta Cyclase Gene (CYC-B) in Tomato Clade
Source: PLoS One. 2016 Apr 12;11(4):e0153333. doi: 10.1371/journal.pone.0153333 (PMC4829152; doi:10.1371/journal.pone.0153333)
Supplement: S9 File — (DOCX) [file pone.0153333.s009.docx]

| **Haplotype** | **No. of accessions** | **Accessions** | **Group** |
| --- | --- | --- | --- |
| Hap_1 | 541 | cv. Arka Vikas*, cv. Heinz_1706 and all other accessions in the study | Lycopersicon |
| Hap_2 | 1 | *S. habrochaites f.glabratum*_LA1362* | Eriopersicon |
| Hap_3 | 1 | *B*_LA3000* | Lycopersicon |
| Hap_4 | 1 | *S. pennellii*_LA0716* | Neolycopersicon |
| Hap_5 | 1 | *S. cheesmaniae*_LA0483* | Lycopersicon |
| Hap_6 | 1 | *S. galapagense*_LA0483 |  |
| Hap_7 | 1 | *S. galapagense*_LA1401 |  |
| Hap_8 | 1 | *S. lycopersicum*_LYC3340 |  |
| Hap_9 | 1 | *S. lycopersicum*_PI129097 |  |
| Hap_10 | 3 | *S. pimpinellifolium*_LYC2798, *S. lycopersicum*var.Cerasiforme cv. Cervil, *S. cheesmaniaex S. lycopersicum*G1.1615(CGN15820) |  |
| Hap_11 | 1 | EC520046* |  |
| Hap_12 | 1 | EC520052* |  |
| Hap_13 | 1 | *S. pimpinellifolium*_LA1578 |  |
| Hap_14 | 2 | *S. lycopersicum*_LYC2962, *S. pimpinellifolium*_LA1584 |  |
| Hap_15 | 1 | cv. KatinkaCherry |  |
| Hap_16 | 1 | WIR3768* |  |
| Hap_17 | 1 | *S. pimpinellifolium*_LA1589* |  |
| Hap_18 | 1 | *S. chilense*_CGN15532 | Eriopersicon |
| Hap_19 | 1 | *S. huaylense*_LA1364 |  |
| Hap_20 | 1 | *S. corneliomuelleri*_LA0118 |  |
| Hap_21 | 1 | *S. peruvianum*_LA1954 |  |
| Hap_22 | 1 | *S. huaylense*_LA1983 |  |
| Hap_23 | 1 | *S. habrochaites f.glabratum*_CGN15791 |  |
| Hap_24 | 1 | *S. neorickii*_LA2133* | Arcanum |
| Hap_25 | 1 | *S. neorickii*_LA2133 |  |
| Hap_26 | 2 | *S. chmielewskii*_LA2663, *S. chmielewskii*_LA2695 |  |
| Hap_27 | 1 | *S. neorickii*_CGN24193 |  |
| Hap_28 | 1 | *S. arcanum*_LA2172 |  |
| Hap_29 | 1 | *S. arcanum*_LA2157 |  |
| Hap_30 | 1 | *S. pennellii*_LYC1831 | Neolycopersicon |
| Hap_31 | 1 | *S. peruvianum*_LA1278 | Eriopersicon |
| Hap_32 | 1 | *S. huaylense*_LA1365 |  |
| Hap_33 | 1 | *S. chilense*_CGN15530 |  |
| Hap_34 | 1 | *S. pennellii*_LA716 | Neolycopersicon |
| Hap_35 | 1 | *S. habrochaites f.glabratum*_PI134418 | Eriopersicon |
| Hap_36 | 1 | *S. habrochaites f.glabratum*_LA1777 |  |
| Hap_37 | 1 | *S. habrochaites f.glabratum*_LA1718 |  |
| Hap_38 | 1 | *S. habrochaites f.glabratum*_CGN15792 |  |
| Hap_39 | 1 | *S. habrochaites f.glabratum*_LA0407 |  |
| Hap_40 | 1 | *S. habrochaites f*.*glabratum*_LYC4 |  |
| Hap_41 | 1 | *Bog*_LA0348* | Lycopersicon |
| Hap_42 | 1 | EC34480* | - |
| Hap_43 | 1 | EC8936* | - |
| Hap_44 | 1 | EC20636* | - |

^*^Analyzed by EcoTILLING and sequencing by Sanger method
