# Supplementary material for: Neofunctionalization of Chromoplast Specific Lycopene Beta Cyclase Gene (CYC-B) in Tomato Clade
Source: PLoS One. 2016 Apr 12;11(4):e0153333. doi: 10.1371/journal.pone.0153333 (PMC4829152; doi:10.1371/journal.pone.0153333)
Supplement: S10 File — (DOCX) [file pone.0153333.s010.docx]

|  | CYCB_Tomato [AK327886.1] | LCYB1_Tomato  [AK319553.1] | LCYB2_Tomato  [AK323472.1] | LCYE_Tomato  [NP_001234337] | CCS_Pepper  [GU122939.1] |
| --- | --- | --- | --- | --- | --- |
| CYCB_Tomato  [AK327886.1] | 100 |  |  |  |  |
| LCYB1_Tomato  [AK319553.1] | 55 | 100 |  |  |  |
| LCYB2_Tomato  [AK323472.1] | 56 | 87 | 100 |  |  |
| LCYE_Tomato  [NP_001234337] | 40 | 37 | 37 | 100 |  |
| CCS_Pepper  [GU122939.1] | 86 | 52 | 58 | 39 | 100 |
