# Supplementary material for: Neofunctionalization of Chromoplast Specific Lycopene Beta Cyclase Gene (CYC-B) in Tomato Clade
Source: PLoS One. 2016 Apr 12;11(4):e0153333. doi: 10.1371/journal.pone.0153333 (PMC4829152; doi:10.1371/journal.pone.0153333)
Supplement: S11 File — Multalin software (http://multalin.toulouse.inra.fr/multalin/multalin.html) (Corpet, 1988) was used for making the alignment. Red color indicates high consensus (>90%) and blue color indicates low consensus (<50%) amino acid residues. The N-terminal region of all the enzymes are highly diverged. (PDF) [file pone.0153333.s011.pdf]

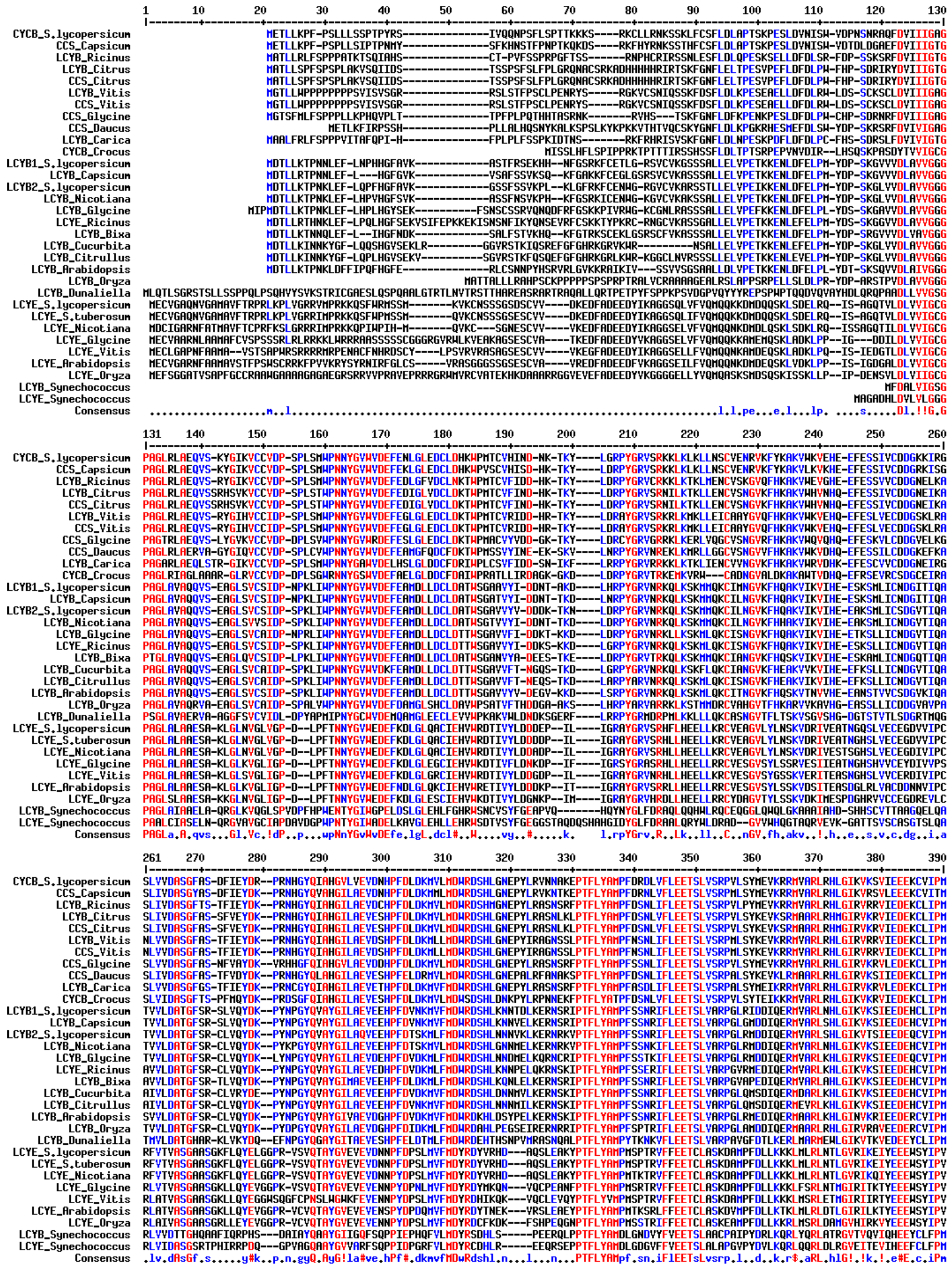

…cont'd in the next page

…cont'd from previous page

```
                     391      400       410       420       430       440       450       460       470       480       490       500       510       520
                     |--------+---------+---------+---------+---------+---------+---------+---------+---------+---------+---------+---------+---------|
 CYCB_S.lycopersicum GGPLPRIPQNVMAIGGNSGIVHPSTGYMVARSMALAPVLAEAIVEGLGS---TRMIRG-----------SQLYHRVWNGLWPLDRRCVRECYSFGMETLLKLDLKGTRRLFDAFFDLDPKYWQGFLSSRL
        CCS_Capsicum GGPLPRIPQNVMAIGGTSGIVHPSSGYMVARSMALAPVLAEAIVESLGS---TRMIRG-----------SQLYHRVWNGLWPSDRRRVRECYCFGMETLLKLDLEGARRLFDAFFDVDPKYWHGFLSSRL
        LCYB_Ricinus GGPLPRIPQTVMAIGGNSGVVHPSTGYMVARTMALAPILADVIAECLGS---TRMIRG-----------RPLYDRVWKGLWPLDKKCTREYYSFGMETLLKLDLKGTRNFFNAFFDLDPYYWEGFLSSRL
         LCYB_Citrus GGPLPVIPQSVMAIGGTSGLIHPATGYMVARTMALAPALADAIAECLGS---TRMIRG-----------RPLHQKVWNGLWPIDRRCNREFYSFGMETLLKLDLKGTRRFFDAFFDLNPYYWHGFLSSRL
          CCS_Citrus GGPLPVIPQSVMAIGGTSGLIHPATGYMVARTMALAPALADAIAECLGS---TRMIRG-----------RPLHQKVWNGLWPIDRRCNREFYSFGMETLLKLDLKGTRRFFDAFFDLNPYYWHGFLSSRL
          LCYB_Vitis GGPXPQIPQSVMAIGGTAGLVHPSTGYMVAKTMALAPVLAETIRECLGS---SRMIRG-----------KPLHHRVWNGLWPSERRFTREFYSFGMETLLKLDLNGTRGFFDAFFDLDPYYWQGFLSSRL
           CCS_Vitis GGPLPQIPQSVMAIGGTAGLVHPSTGYMVAKTMALAPVLAETIRECLGS---SRMIRG-----------KPLHHRVWNGLWPSERRFTREFYSFGMETLLKLDLNGTRGFFDAFFDLDPYYWQGFLSSRL
         CCS_Glycine GGPLPRIPQEVMAIGGTSGVVHPSTGYMVARTMAVAPVVAFAITQCLGS---TRMIRG-----------KQLHDKVWNSMWPIENRLVREFYSFGMETLLKLDLNGSRSFFDAFFNLKPYYWQGFLSSRL
          CCS_Daucus GGPLPRTPQDVVAIGGSSGIVHPSTGYMVARTLALAPVLADAIAECLGS---TRMIRG-----------SSLYHRVWNGLWPIESKCTREFYSFGMETLLKLDLHGTRNFFDAFFDLDPHYWQGFLSSRL
         LCYB_Carica GGPLPKIPQNVMAIGGNSGIVHPSTGYMVARTLALAPVVADAIAECLGS---TRMIRG-----------RPLHYRVWKELWPIERKYAREFYCFGMETLLKLDLNGSRRFFDAFFGLDPDYYHGFLSSRL
         CYCB_Crocus GGPLPMIPQSVMGFGGTAGLVHPATGYSVARALRAAPVVVDAMAELLGS---TRMVRG-----------RQLQQRIWSSLWTAEMKGEREFYRFGMETLLKMDLEGTGGL-HAFFRSGASLLARVLSSGC
LCYB1_S.lycopersicum GGPLPVLPQRVVGIGGTAGMVHPSTGYMVARTLAAAPVVANAIIQYLGS-E-RS-HSG-----------NELSTAVWKDLWPIERRRQREFFCFGMDILLKLDLPATRRFFDAFFDLEPRYWHGFLSSRL
       LCYB_Capsicum GGPLPVLPQRVVGIGGTAGMVHPSTGYMVARTLAAAPVVANAIIQYLSS-E-RS-HSG-----------DELSAAVWKDLWPIERRRQREFFCFGMDILLKLDLPATRRFFDAFFDLEPRYWHGFLSSRL
LCYB2_S.lycopersicum GGPLPVIPQRVVGIGGTAGMVHPSTGYMVARTLAAAPVVANAIVQYLGS-D-KD-HLG-----------NELSASVWKDLWPIERRRQREFFCFGMDILLKLDLSATRRFFDAFFDLEPRYWHGFLSSRL
      LCYB_Nicotiana GGSLPVIPQRVVGTGGTAGLVHPSTGYMVARTLAAAPVVANAIIHYLGS-E-KD-LLG-----------NELSAAVWKDLWPIERRRQREFFCFGMDILLKLDLPATRRFFDAFFDLEPRYWHGFLSSRL
        LCYB_Glycine GGPLPVLPQRVVGIGGTAGMVHPSTGYMVARTLAAAPIVANSIVQCLGS-D-RG-FSG-----------GETSAQVWKDLWPIQRRRQREFFCFGMDILLKLDLPGTRRFFDAFFDLEPHYWHGFLSSRL
        LCYE_Ricinus GGPLPVLPQRVVGIGGTAGMVHPSTGYMVARTLAAAPVVANSIVQYLGS-S-RS-LSG-----------TELSAEVWKDLWPIERRRQREFFCFGMDILLKLDLQATRRFFDAFFDLEPHYWHGFLSSRL
           LCYB_Bixa GGPLPVLPQRVLGIGGTAGMVHPSTGYMVARTLAAAPLVANSIVFYSSS-DHNS-ASG-----------DDLAAQIWKELWPIERRRQREFFCFGMDILLKLDLQATRRFFDAFFDLEPHYWHGFLSSRL
      LCYB_Cucurbita GGQLPVLPQRVVGIGGTAGMVHPSTGYMVARTLAAAPIVAGAIVRCLGS-DGR--FRG-----------DEISSEVWRDLWPIERRRQREFFCFGMDILLKLDLKGTRRLFDAFFDLEPHYWHGFLSSRL
      LCYB_Citrullus GGPLPVLPQRVVGIGGTAGMVHPSTGYMVARTLAAAPIVASAIVRCLGS-DGR--FRG-----------DAISSEVWKDLWPIERRRQREFFCFGMDILLKLDLNGTRRFFDAFFDLEPRYWHGFLSSRL
    LCYB_Arabidopsis GGPLPVLPQRVVGIGGTAGMVHPSTGYMVARTLAAAPIVANAIVRYLGSPSSNS-LRG-----------DQLSAEVWRDLWPIERRRQREFFCFGMDILLKLDLDATRRFFDAFFDLQPHYWHGFLSSRL
          LCYB_Oryza GGPLPVLPQRVVGIGGTAGMVHPSTGYMVARTLATAPIVADAIVRFLDTGSGDSAFAG-----------DALSAEVWRELWPAQRRRQREFFCFGMDILLKLDLDGTRRFFDAFFDLEPRYWHGFLSSRL
     LCYB_Dunaliella GGALPQHPQRVLGIGGTAGMVHPSTGFMMTRMLGSAPVVADAIVDQLSRPTDKATTAGAARQLVTEQGAEEMAAAVWQAAWPVERIRQRIFMEFGMEVLLSLNLQQTRDFFAAFFSLSDFHWQGFLSSRL
 LCYE_S.lycopersicum GGSLPNTEQKTLAFGAAASMVHPATGYSVVRSLSEAPKCASVLANILRQH-YSKNMLTSS-S------IPSISTQAWNTLWPQERKRQRSFFLFGLALILQLDIEGIRSFFRAFFRVPKWMWQGFLGSSL
    LCYE_S.tuberosum GGSLPNTEQKTLAFGAAASMVHPATGYSVVRSLSEAPKCASVLANILRQN-HSKNMLTSS-S------TPSISTQAWNTLWPQERKRQRSFFLFGLALILQLDIEGIRSFFRAFFRVPKWMWQGFLGSSL
      LCYE_Nicotiana GGSLPNTEQKTLAFGAAASMVHPATGYSVVRSLSEAPKCASVLANILRQN-HVKNMLTSS-S------TTSISTQAWNTLWPQERKRQRSFFLFGLALILQLDIEGIRSFFRAFFLVPKWMWQGFLGSSL
        LCYE_Glycine GGSLPNTEQKNLAFGAAASMVHPATG--MTSSLSEAPKYASVIATILKDG-HAKDIITQERR------KENLSMQAWNTLWPQERKRQRAFFLFGLALILQLDIEGIRTFFRTFFCLPDWMWQGFLGSSL
          LCYE_Vitis GGSLPNTEQKNLAFGAAASMVHPATGYSVVRSLSEAPKYASVIANILKRS-HSKDIFTRGRS------TGNIPMQAWNTLWPQERKRQRSFFLFGLALILELDIEGIRTFFHTFFRLPNWMWQGFLGSTL
    LCYE_Arabidopsis GGSLPNTEQKNLAFGAAASMVHPATGYSVVRSLSEAPKYASVIAEILREE-TTKQI------------NSNISRQAWDTLWPPERKRQRAFFLFGLALIVQFDTEGIRSFFRTFFRLPKWMWQGFLGSTL
          LCYE_Oryza GGSLPNTDQKNLAFGAAASMVHPATGYSVVRSLSEAPRYASVISDILRNRVYPGEYLPGTSQ------SSSPSMLAWRTLWPQERKRQRSFFLFGLALIIQLNNEGIQTFFETFFRLPKWMWRGFLGSTL
  LCYB_Synechococcus NLPLPDLTQSVVGFGGAASMVHPASGYMVGALLRRAPDLANAIAAGLNA---SSSLTT-----------AELATQAWRGLWPTEKIRKHYIYQFGLEKLMRFSEAQLNHHFQTFFGLPKEQWYGFLTNTL
  LCYE_Synechococcus NLPLPDRNQPVLAFGGAASMVHPASGYMVGALLRRGPALAQALAEAI-A---NQSLGS-----------AALAQRGWQALWPMELVLRHQLYQFGLGRLMGFNEALLRTHFATFFSLPREEWFGFLTNTL
           Consensus ggpLP..pQ.v.aiGgtagmVHPstGYmVart$a.AP..A.ai...Lgs........g....      ...l...vW..LWP.#r.rqref%.FG$..l$kl#l.gtr.fFdaFFdl.p.yW.GFLssrL

                     521      530       540       550       560       570       580       590 594
                     |--------+---------+---------+---------+---------+---------+---------+---|
 CYCB_S.lycopersicum SVKELGLLSLCLFGHGSNMTRLDIVTKCPLPLVRLIGNLAIESL
        CCS_Capsicum SVKELAVLSLYLFGHASNLARLDIVTKCTVPLVKLLGNLAIESL
        LCYB_Ricinus SLGQLALLSLSLFGNASNSSRFDIVTKCPLPLVRMMNNLALETI
         LCYB_Citrus SLAELAGLSLSLFGHASNSSRLDIVTKCPVPLVKMMGNLALETI
          CCS_Citrus SLAELAGLSLSLFGHASNSSRLDIVTKCPVPLVKMMGNLALETI
          LCYB_Vitis SPRELALLSLSLFGRASNPSRFDIITKCPAPLVKMMGKLAFETI
           CCS_Vitis SPRELALLSLSLFGRASNPSRFDIITKCPAPLVKMMGKLAFETI
         CCS_Glycine TLNELLWLSISLFGHASNPSRFDIVTKCPVPMAKMVGNIALEYIG
          CCS_Daucus SLKELAMLSLSLFGHASNSSKMDIVTKCPAPLVKMLGNLAVETI
         LCYB_Carica SLRELFGLSLSLFIRASNSSRMDIVTNCPAPLIRMVGNLALQPF
         CYCB_Crocus RFRRLAGLCLSLFGHASNSSRLDIVTKCPAPLARMVGNLALQAV
LCYB1_S.lycopersicum FLPELIVFGLSLFSHASNTSRFEIMTKGTVPLVNMINNLLQDKE
       LCYB_Capsicum FLPELIVFGLSLFSHASNTSRLEIMTKGTLPLVHMINNLLQDKE
LCYB2_S.lycopersicum FLPELMFFGLSLFSHASNTSRLEIMTKGTFPLVTMINNLLKDTE
      LCYB_Nicotiana YLPELIFFGLSLFSRASNTSRIEIMTKGTLPLVNMINNLLQDTE
        LCYB_Glycine FLPELIFFGLSLFSYASNTSRIEIMAKGTLPLVNMINNLVKDTE
        LCYE_Ricinus FLPELIFFGLSLFSHASNTSRLEIMAKGTLPLVNMVNNLIQDSE
           LCYB_Bixa FLPELV-FGLSLFSHASNTSRVEIMAKGTIPLVNMINNLLQDKD
      LCYB_Cucurbita FLPELILFGLSLFSHASNASRVEIMAKGTPSLVNMIGNLVKDRD
      LCYB_Citrullus FLPELLLFGLSLFSHASNASRLEIMAKGTPSLVNMIGNLVKDRD
    LCYB_Arabidopsis FLPELLVFGLSLFSHASNTSRLEIMTKGTVPLAKMINNLVQDRD
          LCYB_Oryza FLPELAMFGLSLFAKASNTSRLEIMAKGTAPLAKMIGNLIQDRDR
     LCYB_Dunaliella SFTQLIGFGLSLFVEASPETRLLLLRLGIPGVVQMLFVLLPTVTGYYKQDTTVKDKKFAHDRAAAASISAAKQQ
 LCYE_S.lycopersicum SSADLMLFAFYMFIIAPNDMRKGLIRHLLSDPTGATLIRTYLTF
    LCYE_S.tuberosum SSADLMLFAFYMFIIAPNDMRRGLIRHLLSDPTGATLIRTYLTF
      LCYE_Nicotiana SSADLMLFAFYMFIIAPNDMRKGLIRHLLSDPTGATMIRTYLTF
        LCYE_Glycine SSTDLVLFAFYMFIIAPNNLRMGLVRHLLSDPTGATMIKTYLTI
          LCYE_Vitis SSADLILFAFYMFILAPNQMRMCLIKHLLSDPTGATMLRTYLTV
    LCYE_Arabidopsis TSGDLVLFALYMFVISPNNLRKGLINHLISDPTGATMIKTYLKV
          LCYE_Oryza SSVDLILFAFYMFTIAPNQMRMNLVRHLLSDPTGSTMIKTYLTL
  LCYB_Synechococcus SLPELIQAMLRLFAQAPNDVRWGLMEQQGRELQLFWQAIAAR
  LCYE_Synechococcus PLPRLMGVMLRLFALSPWELRRGLVLGAAQDQAPRF
           Consensus sl.eL..f.ls$F..asn.sR..i..k...pl..m..nl......
```
